# The STAT3/SETDB2 axis dictates NF-**κ**B–mediated inflammation in macrophages during wound repair

**DOI:** 10.1172/jci.insight.179017

**Published:** 2024-10-22

**Authors:** Kevin D. Mangum, Aaron denDekker, Qinmengge Li, Lam C. Tsoi, Amrita D. Joshi, William J. Melvin, Sonya J. Wolf, Jadie Y. Moon, Christopher O. Audu, James Shadiow, Andrea T. Obi, Rachael Wasikowski, Emily C. Barrett, Tyler M. Bauer, Kylie Boyer, Zara Ahmed, Frank M. Davis, Johann Gudjonsson, Katherine A. Gallagher

**Affiliations:** 1 Section of Vascular Surgery, Department of Surgery;; 2 Department of Microbiology and Immunology;; 3 Department of Dermatology;; 4 Department of Computation Medicine and Bioinformatics; and; 5 Department of Biostatistics, University of Michigan, Ann Arbor, Michigan, USA.

**Keywords:** Inflammation, Transcription

## Abstract

Macrophage transition from an inflammatory to reparative phenotype after tissue injury is controlled by epigenetic enzymes that regulate inflammatory gene expression. We have previously identified that the histone methyltransferase SETDB2 in macrophages drives tissue repair by repressing NF-κB–mediated inflammation. Complementary ATAC-Seq and RNA-Seq of wound macrophages isolated from mice deficient in SETDB2 in myeloid cells revealed that SETDB2 suppresses the inflammatory gene program by inhibiting chromatin accessibility at NF-κB–dependent gene promoters. We found that STAT3 was required for *SETDB2* expression in macrophages, yet paradoxically, it also functioned as a binding partner of SETDB2 where it repressed SETDB2 activity by inhibiting its interaction with the NF-κB component, RELA, leading to increased RELA/NF-κB–mediated inflammatory gene expression. Furthermore, RNA-Seq in wound macrophages from STAT3-deficient mice corroborated this and revealed STAT3 and SETDB2 transcriptionally coregulate overlapping genes. Finally, in diabetic wound macrophages, *STAT3* expression and STAT3/SETDB2 binding were increased. We have identified what we believe to be a novel STAT3/SETDB2 axis that modulates macrophage phenotype during tissue repair and may be an important therapeutic target for nonhealing diabetic wounds.

## Abstract 2

The histone methyltransferase SETDB2 limits inflammation in wound macrophages. STAT3 is required for SETDB2 expression, yet inhibits the RelA-SETDB2 interaction, leading to increased inflammation.

## Graphical abstract

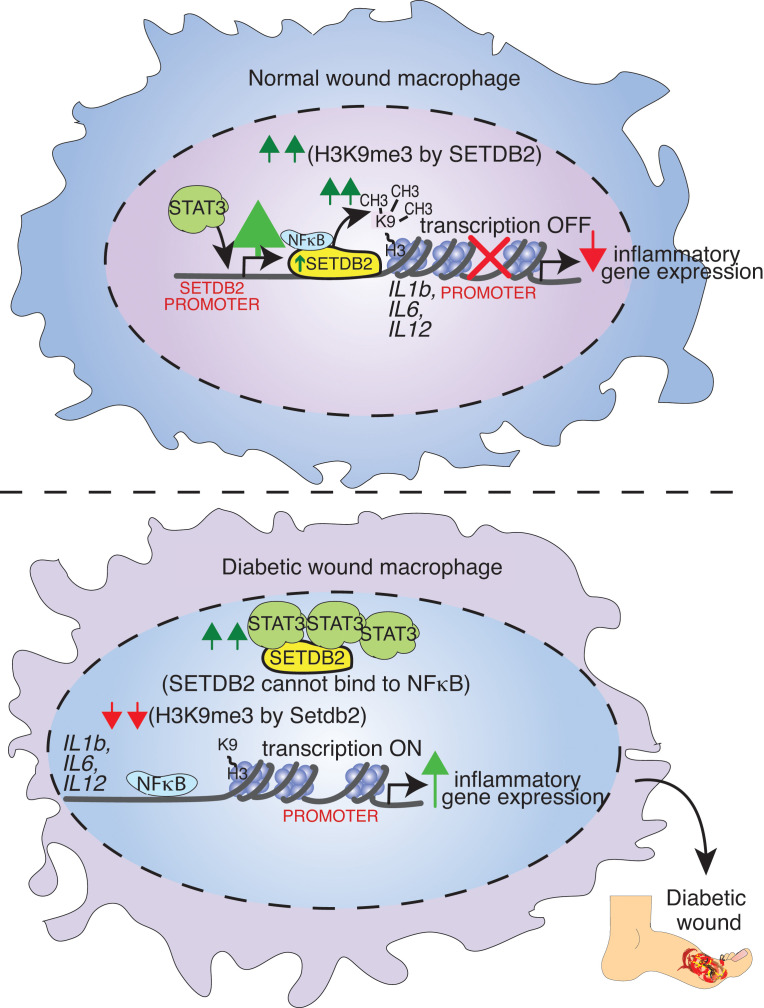

## Introduction

Wound healing is characterized by 4 synchronous stages (hemostasis, inflammation, proliferation involving reepithelialization and contraction, and remodeling), and coordinated progression through each of these stages is essential for normal tissue repair (1, 2). During the inflammatory phase, macrophages exhibit increased expression and production of inflammatory cytokines that leads to phagocytosis and clearance of debris and also aids in the recruitment of other cells necessary for repair (3–5). After resolution of the initial inflammation, macrophages at the wound site transition to a reparative phenotype that is defined by increased expression of antiinflammatory mediators, and proinflammatory macrophages are replaced by new reparative macrophages, which sets the stage for fibroblast functions in the proliferative phase (2, 4, 6–16). The macrophage switch to a reparative phenotype is crucial for wound healing to progress from the inflammatory to the proliferative phase (2, 4, 6–15). The factors that control these transitions are not completely clear; however, our group and others have identified that chromatin-modifying enzymes (CMEs) that regulate the accessibility of transcription factors (TFs) to gene promoters play a critical role in controlling cell phenotype (17–22). In pathologic processes that result in nonhealing, such as occurs in type 2 diabetes (T2D), macrophages fail to transition to a reparative phenotype and remain in a chronic low-grade inflammatory state (10, 12, 18, 23). Related to this, our group has previously identified the histone methyltransferase SETDB2 — which trimethylates H3K9 at gene promoters, resulting in reduced inflammatory gene transcription — is decreased in diabetic wound tissue, leading to increased inflammatory gene expression and a sustained inflammatory response (21, 22). Although we previously identified that SETDB2 is upregulated in normal wound macrophages via an IFN-β/JAK/STAT mechanism, the specific downstream protein-protein interactions and precise mechanism by which SETDB2 results in chromatin accessibility and TF binding remain unknown. Identification of these specific interactions are important from a translational standpoint as the identification of a cell-specific target further downstream to control cell phenotype in tissue repair would be more targetable and have fewer off-target side effects.

The NF-κB signaling pathway is a driver inflammation in macrophages following tissue injury (24). The NF-κB family of TFs is composed of 5 members, or subunits, including RELA (p65), Rel (cRel), RelB, Nfkb1 (p50/p105), and Nfkb2 (p52/p100) (25). Distinct combinations between the different NF-κB components in the form of hetero- and homodimers dictate unique downstream transcriptional functions. Activation of NF-κB signaling by different upstream cytokines, pathogens, growth factors, etc. induces nuclear translocation of the different NF-κB subunits, where they can interact in unique combinatorial ways to regulate gene expression (26). RELA (p65) is one of the major NF-κB subunits activated by proinflammatory cytokines, and while it can directly bind DNA to modulate gene expression, it can also interact with other TFs to either activate or repress inflammatory gene expression (27, 28). Specifically, in cancer cells, RELA and STAT3 physically interact, thereby integrating the NF-κB and JAK/STAT signaling pathways (24, 29). Additionally, STAT3 activation has been found to activate RELA (24, 30). While the transcriptional interaction between RELA and STAT3 has been examined in the context of cancer, it is unclear whether this same mode of regulation exists in the context of macrophage-mediated inflammation during wound healing. Furthermore, we have previously shown that SETDB2 is a downstream transcriptional target of the IFN-β pathway, and IFN-β both increases *SETDB2* expression and SETDB2 enrichment (along with associated H3K9me3) at NF-κB–dependent promoter regions within inflammatory genes (22). However, the specific transcriptional components involved in regulating SETDB2 at the NF-κB–dependent promoter region to control macrophage phenotype have not been identified.

Here, using both human tissue and murine transgenic models, we found that STAT3 interacts with SETDB2, regulating its ability to modulate inflammation in macrophages. Analysis of RNA-Seq and ATAC-Seq of wound macrophages isolated from our *Setdb2^fl/fl^ Lyz2^Cre^* mice with a myeloid cell–specific deletion of *Setdb2* revealed that SETDB2 repressed the inflammatory gene program by limiting chromatin accessibility at NF-κB–dependent gene regions. Using a combination of pharmacologic and siRNA-mediated approaches, we demonstrated that STAT3 physically interacted with SETDB2 to inhibit the SETDB2/NF-κB interaction, resulting in increased NF-κB–dependent gene expression. Interestingly, we also found that STAT3 was required for *SETDB2* transcription. Thus, we identified a paradoxical relationship between STAT3 and SETDB2 in which STAT3 is required for *SETDB2* expression but also limits its activity at the protein-protein level. As a translational corollary, *Stat3^fl/fl^ Lyz2^Cre^* mice with a *Stat3* gene deletion specifically in myeloid cells exhibited improved early wound healing. Stat3-deficient wound macrophages isolated from these mice revealed that *STAT3* expression is dynamic throughout wound healing and serves to coregulate the inflammatory gene program with SETDB2, providing a mechanism for the tightly regulated control of macrophage phenotype. Furthermore, diabetic wound macrophages exhibited increased expression of *STAT3* in murine and human wound samples as well as enhanced binding of STAT3 to SETDB2. We observed that this resulted in an imbalance of STAT3, leading to dysregulated STAT3/SETDB2 binding and an aberrant increase in NF-κB–mediated gene expression. Thus, inhibiting STAT3 at specific time points and preventing its binding to SETDB2 may therefore represent a promising therapeutic target for impaired diabetic wound healing.

## Results

### SETDB2 is enriched at NF-κB–dependent gene promoters in human and murine wound macrophages.

**Figure 1:**
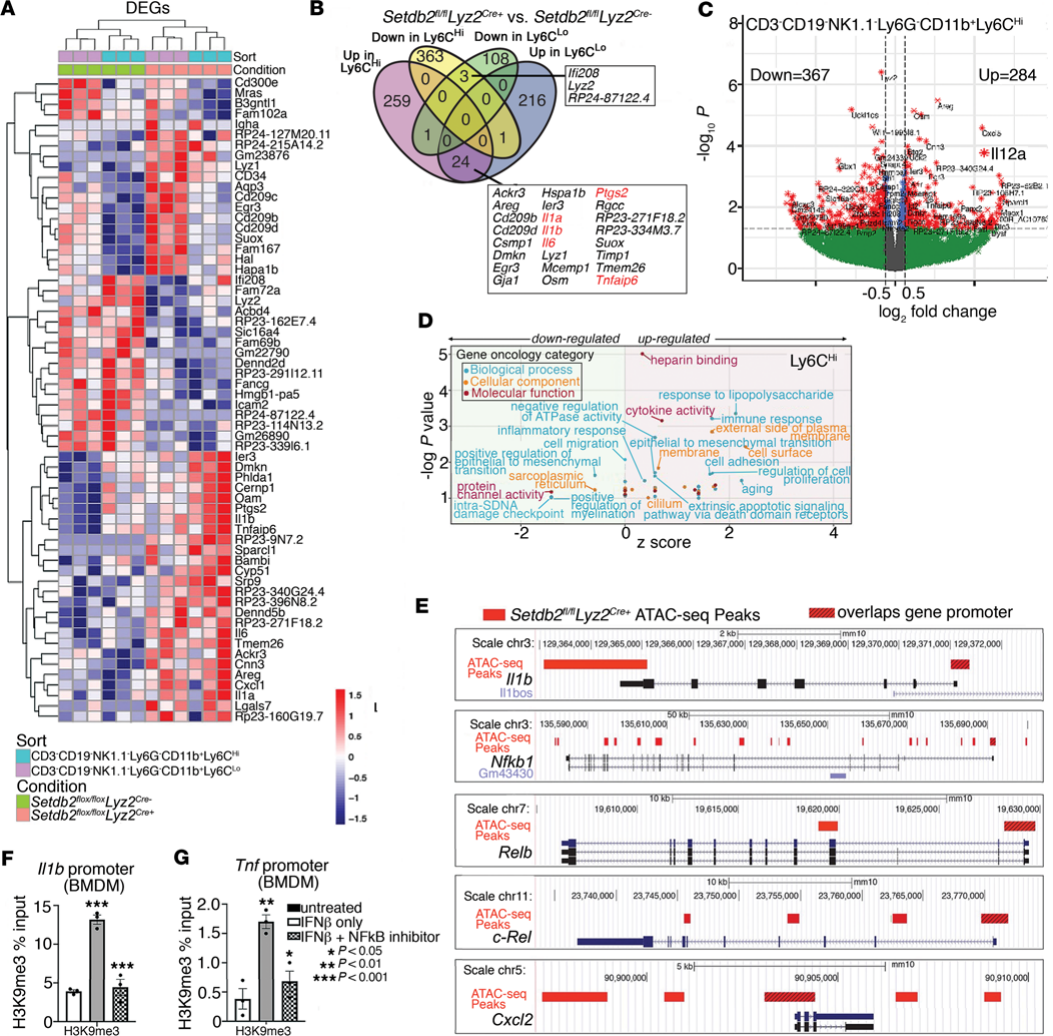
SETDB2 is enriched at NF-κB–dependent gene promoters in murine wound macrophages. Wound macrophages were isolated from *Setdb2^fl/fl^ Lyz2*^Cre^ *mTmG* murine wounds on day 5 after wounding, sorted into CD3^–^CD19^–^NK1.1^–^Ly6G^–^CD11b^+^Ly6C^hi^ and CD3^–^CD19^–^NK1.1^–^Ly6G^–^CD11b^+^Ly6C^lo^ populations, and then analyzed for gene expression by RNA-Seq and ATAC-Seq. (**A**) Heatmap of DEGs in wound macrophages isolated from *Setdb2^fl/fl^ Lyz2*^Cre^ *mTmG* murine wounds on day 5 after wounding. Wound macrophages were sorted into CD3^–^CD19^–^NK1.1^–^Ly6G^–^CD11b^+^Ly6C^hi^ and CD3^–^CD19^–^NK1.1^–^Ly6G^–^CD11b^+^Ly6C^lo^ populations and then analyzed for gene expression by RNA-Seq (12–15 mice per group, *n* = 3–5 independent experiments). (**B**) Venn diagram comparing upregulated and downregulated genes from RNA-Seq of wound macrophages (CD3^–^CD19^–^ NK1.1^–^Ly6G^–^CD11b^+^Ly6C^hi^ and CD3^–^CD19^–^NK1.1^–^Ly6G^–^CD11b^+^Ly6C^lo^) isolated from *Setdb2^fl/fl^ Lyz2*^Cre^ *mTmG* mouse wounds on day 5 after wounding. (**C**) Volcano plot of transcriptomic profiles from DEGs in CD3^–^CD19^–^NK1.1^–^Ly6G^–^CD11b^+^Ly6C^hi^ wound macrophages from *Setdb2^fl/fl^ Lyz2*^Cre^ *mTmG* mice. (**D**) GO analysis of DEGs in CD3^–^CD19^–^NK1.1^–^Ly6G^–^CD11b^+^Ly6C^hi^ wound macrophages from *Setdb2^fl/fl^ Lyz2*^Cre^ *mTmG* mice. (**E**) Inflammatory gene promoter regions differentially regulated by SETDB2 as determined by ATAC-Seq of wound macrophages (CD3^–^CD19^–^NK1.1^–^Ly6G^–^CD11b^+^Ly6C^+^ GFP^+^) isolated on day 5 from *Setdb2^fl/fl^ Lyz2*^Cre^ *mTmG* mice. Red bars indicate ATAC-Seq peaks relative to Cre-negative controls. Hashed red bars designate peaks that overlap promoter regions of listed genes. (**F** and **G**) H3K9me3 ChIP qPCR at the *Il1b* and *Tnf* promoters in murine BMDMs unstimulated or treated with IFN-β (10 U/mL; 8.5 ng/mL) or IFN-β and NF-κB inhibitor, BAY 11-7082 (10 μM). Data are representative of *n* = 3–5 independent experiments, with 12–15 mice per group per experiment. *P < 0.05, **P < 0.01, ***P < 0.001. Data are presented as the mean ± SEM. Two-tailed Student’s *t* test was used for comparison of 2 groups. For comparison among multiple groups, 2-way ANOVA followed by Newman-Keuls post hoc test was used.

In order to examine the downstream genes controlled by SETDB2 in wound macrophages and elucidate the exact transcriptional targets of SETDB2 that control macrophage phenotype, we performed FACS for CD3^–^CD19^–^Ly6G^–^CD11b^+^Ly6C^hi^GFP^+^ and CD3^–^CD19^–^Ly6G^–^CD11b^+^Ly6C^lo^GFP^+^ cells from murine wounds in *Setdb2^fl/fl^ Lyz2*^Cre+^ *mTmG* mice and *Setdb2^fl/fl^ Lyz2*^Cre–^ *mTmG* littermate controls.In this system, successful genetic recombination leads to simultaneous excision of the Tomato reporter and activation of the GFP reporter, resulting in GFP expression in cells that have undergone successful Cre-mediated deletion of the floxed gene. Given that we had identified previously that *SETDB2* exhibits its highest expression in wound macrophages at day 5 after wounding, we isolated day 5 wound macrophages from the *Setdb2^fl/fl^ Lyz2*^Cre^ *mTmG* mice to identify maximal alterations in the downstream transcriptional signatures in macrophages after *SETDB2* deletion (22). Because Ly6C is a well-established surface marker designating the inflammatory macrophage subset, we further used FACS to separate the CD3^–^CD19^–^Ly6G^–^CD11b^+^GFP^+^ population into CD3^–^CD19^–^Ly6G^–^CD11b^+^GFP^+^Ly6C^hi^ (Ly6C^hi^) and CD3^–^CD19^–^Ly6G^–^CD11b^+^GFP^+^Ly6C^lo^ (Ly6C^lo^) subpopulations in order to delineate transcriptional differences based on inflammatory subtype. RNA-Seq analysis was performed in Ly6C^hi^ and Ly6C^lo^ subpopulations, and we identified gene expression profiles of these subtypes and examined how SETDB2 affected gene expression in these populations. As expected, we observed significant differences in the genes differentially expressed between Ly6C^hi^ and Ly6C^lo^, which were further altered by SETDB2 deficiency (Figure 1A). Additionally, we found SETDB2 controlled expression of inflammatory genes that play a key role in wound repair, including *Il1b*, *Il6*, *Il12*, and *Tnf*, and these genes were further increased in the Ly6C^hi^ population by SETDB2 deletion (Figure 1, B and C). A complete list of differentially expressed genes (DEGs) is listed in Supplemental Table 1 (supplemental material available online with this article; https://doi.org/10.1172/jci.insight.179017DS1). Importantly, many of the DEGs identified are well-known downstream targets of the TNF-α and IFN-β signaling pathways, which play critical roles in regulating inflammation during important biological processes, including wound healing (22, 31, 32). In support of SETDB2 regulating the inflammatory phenotype, these proinflammatory genes were more enriched in the Ly6C^hi^ macrophage population, which exhibits an increased inflammatory signature compared with the Ly6C^lo^ macrophage subtype. To further delineate the downstream pathways regulated by SETDB2, we performed gene ontology (GO) analysis on the DEGs, and it supported the unique transcriptional signatures between Ly6C^hi^ and Ly6C^lo^ subpopulations in wound macrophages (Figure 1D and Supplemental Figures 1–3). Additionally, pathways including immune response, response to LPS, and cytokine activity were also upregulated in Setdb2-deficient Ly6C^hi^ macrophages, further supporting the role of SETDB2 in limiting the inflammatory phenotype of this subpopulation. Furthermore, pathway analysis of the Setdb2-deficient Ly6C^hi^ macrophage subpopulation revealed increased activation of IL-1R and NF-κB signaling, providing additional support for SETDB2 in negatively regulating these inflammatory pathways (Supplemental Figure 4). Because changes in gene expression may not necessarily reflect alterations in chromatin structure due to SETDB2 deletion, we next performed ATAC-Seq on FACS-isolated CD3^–^CD19^–^Ly6G^–^CD11b^+^Ly6C^+^GFP^+^ cells from wounds of *Setdb2^fl/fl^ Lyz2^Cre+^* and Cre-negative controls to interrogate chromatin accessibility in Setdb2-deficient wound macrophages, with particular attention given to the inflammatory-related genes identified by RNA-Seq in Figure 1A. Our ATAC-Seq analysis identified increased chromatin accessibility within inflammatory genes (*Il1b*, *Nfkb1*, *Relb*, *Rel*, *Cxcl2*) in Setdb2-deficient Ly6C^hi^ wound macrophages compared with Setdb2-competent Ly6C^hi^ cells (Figure 1E) (Supplemental Table 2). There were 2 additional noteworthy findings from these initial unbiased sequencing results. First, as we had previously shown (22), SETDB2 leads to silencing of inflammatory genes by promoting a closed chromatin configuration via trimethylation of H3K9. Second, SETDB2 regulates chromatin accessibility in NF-κB–binding regions in inflammatory gene promoters, which again corroborated previous findings that SETDB2 transcriptionally targeted NF-κB–bound promoter regions. Since we identified that SETDB2 specifically regulates NF-κB–binding regions in the promoter regions of inflammatory genes, we sought to more specifically examine the interactions between SETDB2 and NF-κB at known NF-κB target genes. Murine BM-derived macrophages (BMDMs) were isolated and treated with IFN-β (10 U/mL; 8.5 ng/mL), which we previously determined to be a potent upstream regulator of SETDB2, with or without an NF-κB inhibitor (BAY 11-7082, 10 μM). ChIP analysis for H3K9me3, the repressive epigenetic mark deposited by SETDB2, at inflammatory gene promoters was then performed. IFN-β resulted in an expected increase in H3K9me3 at the *Il1b* and *Tnf* promoters, and this H3K9 trimethylation was reversed by treatment with an NF-κB inhibitor (Figure 1, F and G). Taken together, these data indicate that SETDB2 represses NF-κB–dependent inflammatory gene expression by regulating chromatin accessibility within these genes, and paradoxically, NF-κB positively regulates SETDB2 function at these gene promoters.

### STAT3 regulates SETDB2 expression in human and murine wound macrophages and is required for normal wound healing.

**Figure 2:**
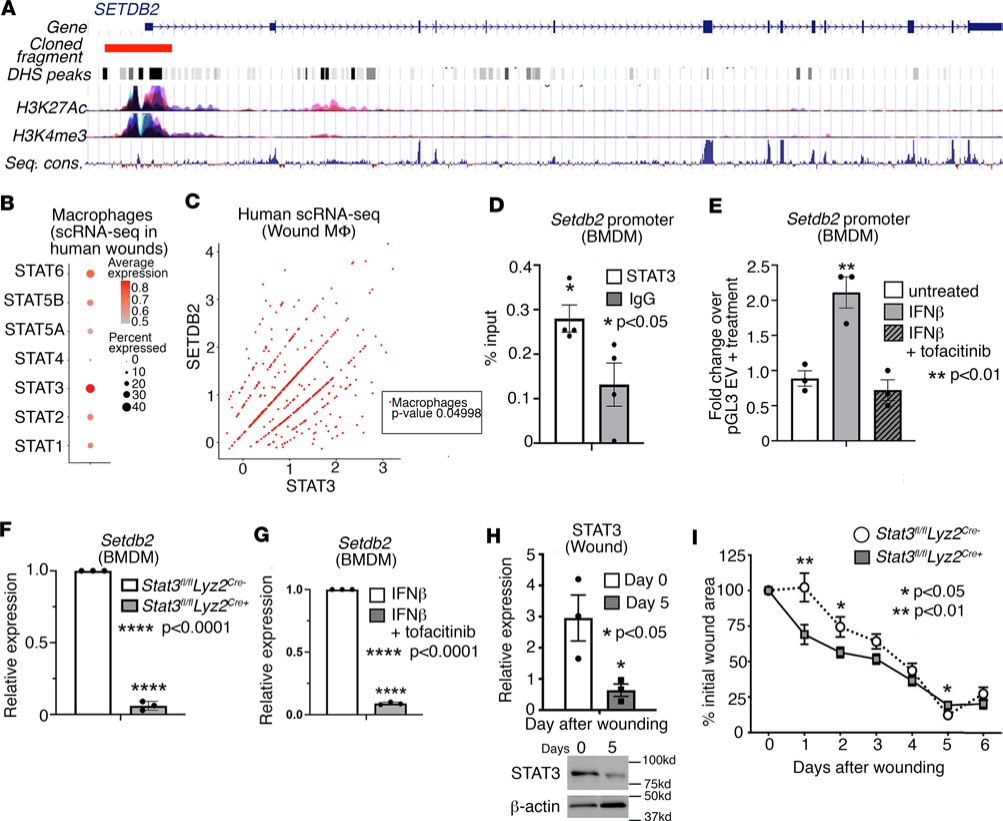
STAT3 is dynamic during wound repair and regulates *SETDB2* in human and murine wound macrophages. (**A**) Gene structure of the human *SETDB2* gene showing cloned fragment aligned with multiple active transcriptional features including DHS peaks, H3K27Ac, H3K4me3, and sequence conservation. (**B**) Dot plot of different STAT members expression from scRNA-Seq data of human wounds (*n* = 10 patients). (**C**) Scatterplot of *SETDB2* and *STAT3* expression in macrophages obtained from scRNA-Seq in human wounds with respective Pearson correlation analysis (*n* = 10 patients). (**D**) ChIP-qPCR for Stat3 binding at the mouse *Setdb2* promoter compared with IgG negative control. (**E**) Luciferase activity of the 3 kb human *SETDB2* promoter cloned into pGL3 and then transfected in BMDMs untreated or treated with IFN-β (10 U/mL; 8.5 ng/mL), or IFN-β plus tofacitinib (100 μM) for 4 hours. (**F**) qPCR analysis of *Setdb2* expression in wound macrophages (CD3^–^CD19^–^NK1.1^–^Ly6G^–^CD11b^+^) isolated from *Stat3^fl/fl^ Lyz2^Cre^* mice on day 5 after wounding compared with Cre^–^ littermate control (*n* = 6 mice per group). (**G**) *Setdb2* expression in BMDMs treated with IFN-β or IFN-β plus tofacitinib (*n* = 6–8 mice per group). (**H**) Representative Western blot and densitometry of murine whole wounds showing decreased levels of STAT3 at day 5 compared with day 0 after wounding (*n* = 3–4 mice at each time point). (**I**) Wound curve analysis in *Stat3^fl/fl^ Lyz2^Cre^* mice compared with Cre^–^ littermate controls all fed a normal diet (*n* = 8–12 mice per group in each experiment). All data are representative of *n* = 3–5 independent experiments. *P < 0.05, **P < 0.01, ****P < 0.0001. Data are presented as the mean ± SEM. Two-tailed Student’s *t* test was used for comparison of 2 groups. For comparison among multiple groups, 2-way ANOVA followed by Newman-Keuls post hoc test was used.

We investigated the upstream transcription mechanisms regulating *SETDB2* expression in macrophages in response to injury. We used a combination of in silico, in vitro, and in vivo approaches to identify TFs regulating expression of *SETDB2*. Because our group has previously identified that STAT proteins regulate *SETDB2* expression, we analyzed the human *SETDB2* promoter for predicted STAT binding sites. Using the publicly accessible database of TF binding, JASPAR, we examined various STAT protein binding sites and found that STAT3 was the most predominant STAT TF predicted to bind to the *SETDB2* promoter. We identified 24 putative STAT3 binding sites within the human *SETDB2* promoter, which also overlapped with multiple features of active transcription including H3K27Ac, H3K4me3, and open chromatin DNase Hypersensitive Sites (DHS), which were all identified using the UCSC Genome Browser (https://genome.ucsc.edu/) (Figure 2A) (33, 34). Although we had previously identified the importance of STAT1 in regulating *SETDB2* in macrophages (22), based on our more recent and robust data of scRNA-Seq of human wounds as well as analysis of TF binding to the *SETDB2* promoter, we determined that STAT3 was the most strongly and abundantly expressed STAT member in wound macrophages (Figure 2B). Additionally, to further examine the relationship between STAT3 and SETDB2, we performed Pearson correlation analysis in macrophages in our scRNA-Seq of skin wounds from patients. We found that *STAT3* and *SETDB2* exhibited a significant and very strong correlation in human wound macrophages (Figure 2C). Next, to confirm Stat3 binding to the *Setdb2* promoter, we isolated murine macrophages and performed ChIP for Stat3. We identified significant Stat3 enrichment at the *Setdb2* promoter over an IgG^–^ control antibody (Figure 2D). To localize the effect of STAT3 function to the *SETDB2* promoter, we cloned a 3 kb fragment of the human *SETDB2* transcription start site into a pGL3 luciferase vector and measured its activity in BMDMs. As shown in Figure 2E, *SETDB2* promoter activity increased 2-fold in BMDMs treated with IFN-β, and this increase was inhibited by treatment with the JAK1/3 inhibitor tofacitinib (100 μM). Furthermore, in vivo wound macrophages (CD3^–^CD19^–^Ly6G^–^CD11b^+^Ly6C^+^) isolated from *Stat3^fl/fl^ Lyz2^Cre+^* mice and Cre-negative littermate controls exhibited decreased *Setdb2* expression (Figure 2F), and treatment of BMDMs with tofacitinib decreased *Setdb2* expression (Figure 2G). Next, to examine STAT3 kinetics throughout normal wound healing, we harvested wound macrophages (CD3^–^CD19^–^NK1.1^–^Ly6G^–^CD11b^+^Ly6C^+^) from mice on days 0 and 5 after wounding and performed Western blotting for STAT3. STAT3 significantly decreased from day 0 to day 5, indicating that it is highly dynamic throughout wound healing (Figure 2H). Finally, to test the role of STAT3 in macrophages during wound repair, we wounded *Stat3^fl/fl^ Lyz2^Cre+^* and Cre-negative controls and measured wound size throughout healing using NIH ImageJ software. Myeloid cell–specific deletion of Stat3 resulted in smaller wounds at earlier time points; however, at day 5, loss of Stat3 in macrophages led to modestly larger wounds, suggesting that Stat3 regulates macrophage phenotype during wound repair in a time-dependent manner (Figure 2I). Taken together, these results suggest that STAT3 is dynamic throughout wound healing, drives *SETDB2* expression in both human and murine wound macrophages during this process, and regulates wound healing in a time-dependent manner.

### STAT3 inhibits the physical interaction between SETDB2 and NF-κB in wound macrophages.

**Figure 3:**
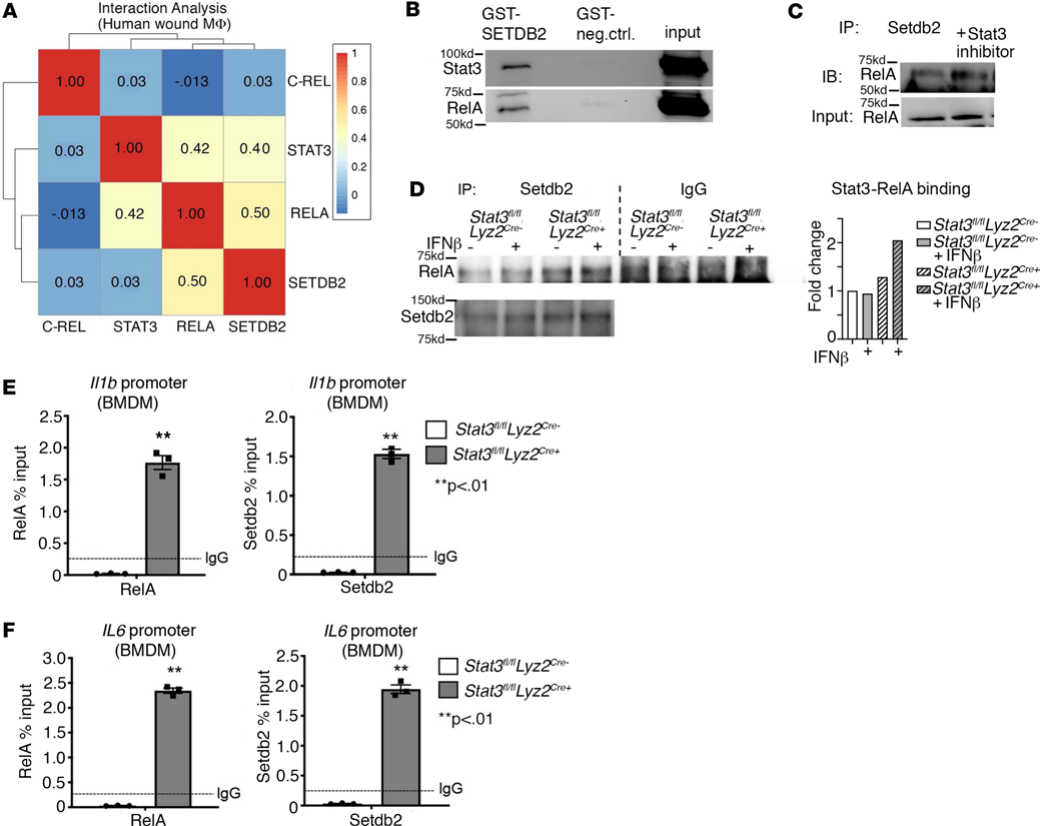
STAT3 inhibits the physical interaction between SETDB2 and NF-κB in wound macrophages. (**A**) Heatmap of Pearson gene correlation analysis results for SETDB2, STAT3, C-REL, and RELA in macrophages obtained from scRNA-Seq in human wounds (*n* = 10 patients). (**B**) Western blot showing Setdb2-interacting proteins purified after incubating GST-SETDB2 with BMDM lysate. (**C**) Western blot of RelA coimmunoprecipitated with Setdb2 from BMDMs treated with Stat3 inhibitor compared with untreated BMDMs. (**D**) Setdb2 immunoprecipitate reactions from BMDMs isolated from *Stat3^fl/fl^ Lyz2^Cre+^* mice and Cre-negative littermate controls that were untreated or treated with IFN-β (10 U/mL; 8.5 ng/mL) for 4 hours were analyzed via Western blot and probed for RelA and Setdb2 (*n* = 4–6 mice per group). (**E** and **F**) ChIP-qPCR on BMDMs isolated from *Stat3^fl/fl^ Lyz2^Cre+^* versus Cre-negative controls for NF-κB (RelA) and Setdb2 at the *Il1b* and *Il6* promoters (*n* = 4–6 mice per group). All data are representative of *n* = 3–5 independent experiments. **P < 0.01. Data are presented as the mean ± SEM. Two-tailed Student’s *t* test was used for comparison of 2 groups. For comparison among multiple groups, 2-way ANOVA followed by Newman-Keuls post hoc test was used.

Since we identified that SETDB2 selectively targeted NF-κB–bound promoter regions and that NF-κB was required for SETDB2 activity at inflammatory gene promoters, we examined the protein-protein interactions between SETDB2 and NF-κB. Hence, we performed an expression-interaction analysis in macrophages from our single-cell RNA-Seq (scRNA-Seq) dataset of human wounds to identify candidate SETDB2 binding partners in a cell type–specific manner. We identified the highest interaction scores between SETDB2, RELA (NF-κB component) (interaction score 0.50), and STAT3 (interaction score 0.40) (Figure 3A). To confirm SETDB2 binding to 1 or more of these TFs, we performed glutathione-*S*-transferase (GST) pulldown assays using recombinant GST-SETDB2 versus a GST-only negative control in BMDM lysate. We identified NF-κB component p65 (RELA) and STAT3 as SETDB2 binding partners (Figure 3B). Notably, RELA and STAT3 exhibited similar binding strengths to SETDB2 relative to their inputs, consistent with interaction scores of 0.50 and 0.40, respectively, in our candidate interaction analysis, further supporting our findings.

Given the similar interaction scores between STAT3 and RELA and equal binding stoichiometry of STAT3 and RELA to GST-SETDB2, we hypothesized that STAT3 perhaps altered binding of RELA and SETDB2. To test this, we first performed Setdb2 immunoprecipitation in BMDMs treated with a Stat3 inhibitor (cryptotanshinone, 1 μM). Stat3 inhibition increased the association between Setdb2 and RelA, suggesting that indeed Stat3 negatively regulated Setdb2/RelA binding (Figure 3C). Additionally, we performed coimmunoprecipitation of endogenous Setdb2 and RelA in BMDMs deficient in Stat3 (*Stat3^fl/fl^ Lyz2*^Cre+^) versus Cre-negative littermate controls. More NF-κB bound to Setdb2 in the Stat3-deficient macrophages compared with controls, confirming our previous findings that Stat3 interfered with the Setdb2/RelA interaction. Notably, in this same set of experiments, IFN-β resulted in a modest increase in NF-κB binding to Setdb2, which is consistent with our RNA-Seq and ATAC-Seq findings of regulation of NF-κB–dependent promoters by Setdb2 and our prior findings that Setdb2 activity is regulated by IFN-β (Figure 3D). To test the effect of Stat3 on Setdb2 and RelA binding in macrophages, we performed ChIP for Setdb2 and RelA in *Stat3^fl/fl^ Lyz2*^Cre+^ or Cre-negative littermate control BMDMs and found increased enrichment of both Setdb2 and NF-κB in Stat3-null macrophages at *Il1b* and *Il6* promoters compared with *Stat3^fl/fl^ Lyz2^Cre–^* macrophages (Figure 3, E and F). We performed this same set of experiments in in vivo wound macrophages (CD3^–^CD19^–^NK1.1^–^Ly6G^–^CD11b^+^) isolated on day 5 and found increased Setdb2 and RelA binding to the *Il1b* promoter in Stat3-deficient macrophages (Supplemental Figure 5). Taken together, these results identify that STAT3 can inhibit NF-κB and SETDB2 binding interactions in wound macrophages, and thus, levels of STAT3 may be critically important for the precise control of inflammatory gene expression by both a direct effect on *SETDB2* expression as well as interference with the SETDB2/NF-κB binding interactions at inflammatory gene promoter sites.

### STAT3 and SETDB2 coregulate inflammatory gene expression in wound macrophages.

**Figure 4:**
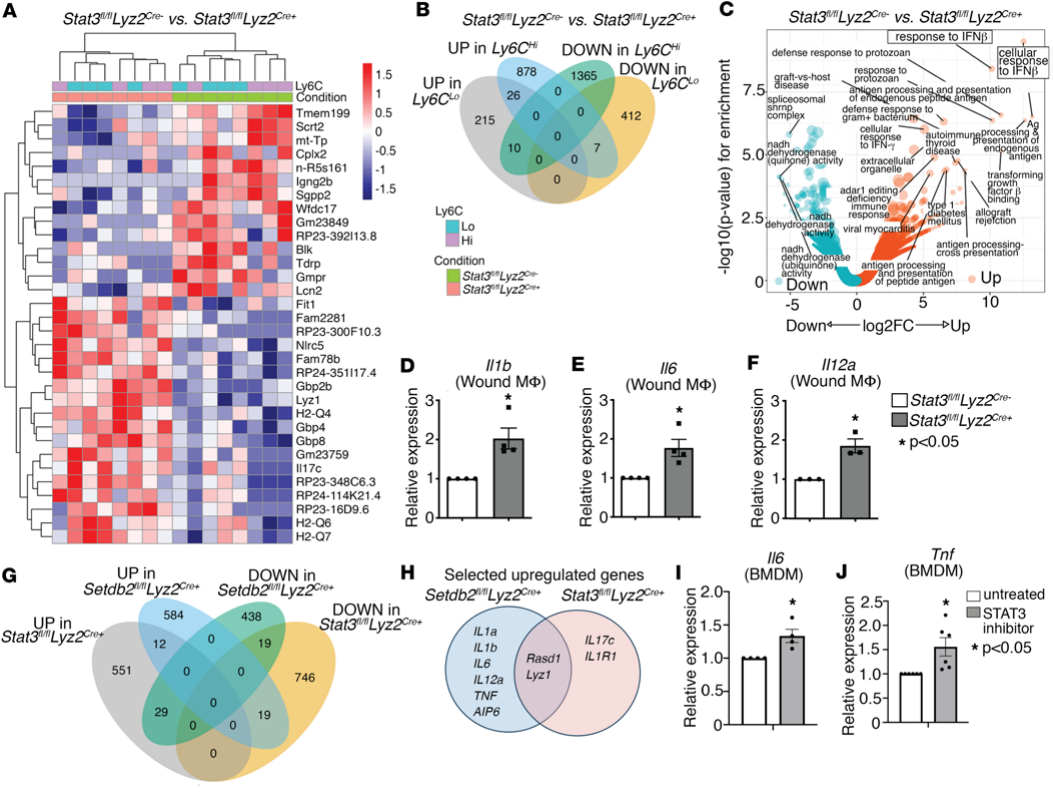
STAT3 and SETDB2 coregulate inflammatory gene expression in wound macrophages. (**A**) Heatmap of DEGs in day 5 wound macrophages (CD3^–^CD19^–^NK1.1^–^Ly6G^–^CD11b^+^) from *Stat3^fl/fl^ Lyz2^Cre^* mice and Cre^–^ littermate controls (*n* = 6 mice per group). (**B**) Diagram comparing overlapping genes among upregulated and downregulated genes from RNA-Seq of *Stat3^fl/fl^ Lyz2^Cre^* wound macrophages (CD3^–^CD19^–^NK1.1^–^Ly6G^–^CD11b^+^). (**C**) Volcano plot of GO analysis for the enriched biological processes in decreased and increased DEGs from *Stat3^fl/fl^ Lyz2^Cre^* wound macrophages. (**D**–**F**) qPCR of *Il1b* (**D**), *Il6* (**E**), and *Il12a* (**F**) in of *Stat3^fl/fl^ Lyz2^Cre^* BMDMs treated with IFN-β (10 U/mL; 8.5 ng/mL) for 6 hours (*n* = 5 mice per group). (**G**) Diagram demonstrating overlapping genes among upregulated and downregulated DEGs from RNA-Seq of *Setdb2^fl/fl^ Lyz2^Cre^* and *Stat3^fl/fl^ Lyz2^Cre^* CD3^–^CD19^–^NK1.1^–^Ly6G^–^CD11b^+^ macrophages isolated from day 5 wounds. (**H**) Venn diagram of overlapping and distinct upregulated inflammatory genes in the CD3^–^CD19^–^NK1.1^–^Ly6G^–^CD11b^+^Ly6C^hi^ population from wounds isolated on day 5 from *Setdb2^fl/fl^ Lyz2^Cre^* and *Stat3^fl/fl^ Lyz2^Cre^* mice. (**I** and **J**) mRNA expression of *Il6* and *Tnf* in BMDMs treated with a STAT3 inhibitor compared with untreated (*n* = 5 mice per group in each experiment). All data are representative of *n* = 3–5 independent experiments. *P < 0.05. Data are presented as the mean ± SEM. Two-tailed Student’s *t* test was used for comparison of 2 groups. For comparison among multiple groups, 2-way ANOVA followed by Newman-Keuls post hoc test was used.

Given the important role of STAT3 in regulating both *SETDB2* expression and SETDB2 activity, we examined the transcriptional targets of STAT3 during wound healing. We selected the day 5 time point after wound healing similar to our earlier RNA-Seq and ATAC-Seq analyses in *Setdb2^fl/fl^ Lyz2^Cre^* mice in order to ensure accurate comparisons between STAT3 and SETDB2 deletion. Wound macrophages were isolated from *Stat3^fl/fl^ Lyz2^Cre+^* and Cre-negative littermate controls day 5 after wounding and then sorted by FACS for CD3^–^CD19^–^Ly6G^–^CD11b^+^Ly6C^+^ and separated into Ly6C^hi^ and Ly6C^lo^ populations. We then performed RNA-Seq on these wound macrophages and found increased expression of genes in critical inflammatory gene pathways, including the IL-17 and IL-1BR pathways, in wound macrophages deficient in Stat3 (Figure 4A and Supplemental Table 3). Additionally, similar to our findings observed in *Setdb2^fl/fl^ Lyz2^Cre+^* wound macrophages (Figure 1), inflammatory transcriptional targets of Stat3 were higher in the Ly6C^hi^ macrophage population, consistent with the proinflammatory macrophage phenotype of this subpopulation. To identify significant regulatory pathways downstream of Stat3, we performed GO analysis of DEGs in *Stat3^fl/fl^ Lyz2^Cre+^* wound macrophages and identified upregulation of genes involved in the IFN-β and TNF-α signaling pathways in the Stat3-deficient macrophages (Figure 4, B and C). Furthermore, we validated our RNA-Seq data and coregulation between Stat3 and Setdb2 by performing quantitative PCR (qPCR) for a subset of proinflammatory genes that were upregulated in *Setdb2^fl/fl^ Lyz2^Cre+^* macrophages. We observed increased expression of inflammatory cytokines including *Il1b*, *Il6*, and *Il12a* in *Stat3^fl/fl^ Lyz2^Cre+^* BMDMs compared with controls (Figure 4, D–F).

Based on our observation that SETDB2 and STAT3 physically interacted and were both involved in the regulation of similar inflammatory genes, we examined whether they regulated overlapping signaling pathways. To address this, we performed GO analysis similar to above but specifically examined for enrichment of genes that were upregulated in both the *Setdb2^fl/fl^ Lyz2^Cre+^* and *Stat3^fl/fl^ Lyz2^Cre+^* wound macrophages. We found a total of 584 genes that were upregulated (compared with 438 downregulated) in Setdb2-deficient macrophages, and there were a total of 551 genes upregulated (compared with 746 downregulated) in Stat3-deficient macrophages (Figure 4G). Out of these, 12 common genes were upregulated and 19 common genes were downregulated in both *Setdb2^fl/fl^ Lyz2^Cre+^* and *Stat3^fl/fl^ Lyz2^Cre+^* macrophages. Similar analysis showed 29 genes that were downregulated in *Setdb2^fl/fl^ Lyz2^Cre+^* but upregulated in *Stat3^fl/fl^ Lyz2^Cre+^*, and there were 19 genes that were upregulated in *Setdb2^fl/fl^ Lyz2^Cre+^* that were downregulated in *Stat3^fl/fl^ Lyz2^Cre+^*. Within only the Ly6C^hi^ population, *Rasd1* and *Lyz1* were upregulated in both *Setdb2^fl/fl^ Lyz2^Cre+^* and *Stat3^fl/fl^ Lyz2^Cre+^* groups (Figure 4H). As additional confirmation of our findings that Stat3 inhibited downstream inflammatory gene expression, we performed pharmacologic inhibition of Stat3 in murine BMDMs by treating with cryptotanshinone (1 μM) for 6 hours. Stat3 inhibition in BMDMs led to increased expression of *Il6* and *Tnf* (Figure 4, I and J). Taken together, these results indicate that SETDB2 and STAT3 coregulate the transcription of proinflammatory genes and that this STAT3/SETDB2 interaction is critical for controlling inflammatory macrophage phenotype during wound healing.

### STAT3 is increased in diabetic wound macrophages during the transition to the reparative phenotype.

**Figure 5:**
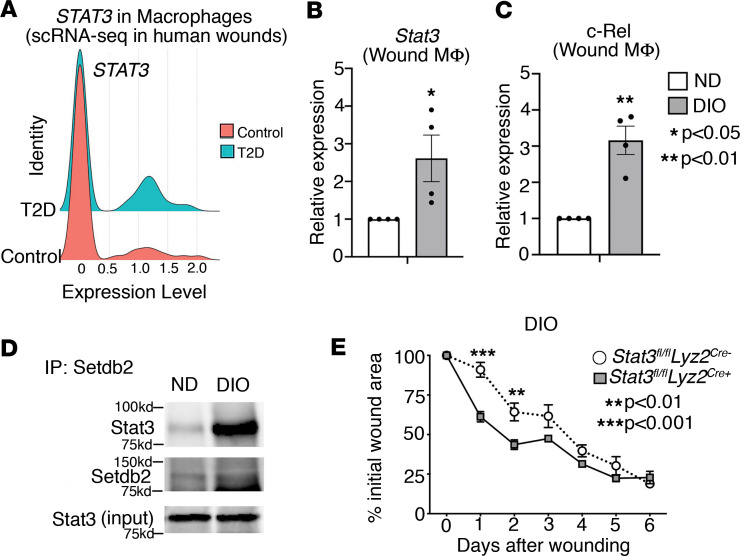
STAT3 is increased in diabetic wound macrophages during the transition to the reparative phenotype. (**A**) scRNA-Seq analysis of *STAT3*, *C-REL*, and *RELA* in macrophages from human wounds isolated from nondiabetic and diabetic patients (*n* = 10 patients). (**B** and **C**) qPCR of *Stat3* and *c-Rel* expression on day 0 and 5 after wounding in ND and DIO murine wound macrophages (CD3^–^CD19^–^NK1.1^–^Ly6G^–^CD11b^+^) treated with IFN-β (10 U/mL; 8.5 ng/mL) for 6 hours ex vivo (*n* = 12–16 mice per group). (**D**) Coimmunoprecipitation of endogenous Setdb2 and Stat3 from ND and DIO whole wounds isolated day 5 after wounding (*n* = 4 mice per group). (**E**) Wound curve analysis in *Stat3^fl/fl^ Lyz2^Cre^* DIO mice compared with Cre^–^ littermate DIO controls (*n* = 8–12 mice per group). All data are representative of *n* = 3–5 independent experiments. *P < 0.05, **P < 0.01, ***P < 0.001. Data are presented as the mean ± SEM. Two-tailed Student’s *t* test was used for comparison of 2 groups. For comparison among multiple groups, 2-way ANOVA followed by Newman-Keuls post hoc test was used.

**Figure 6:**
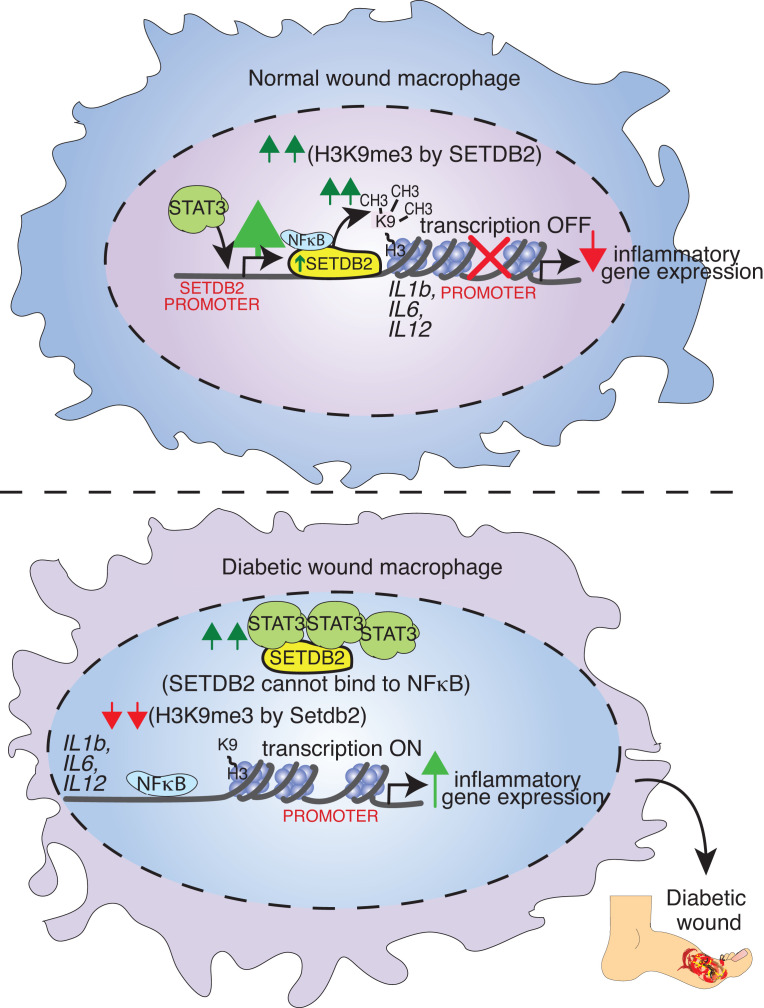
Schematic of the STAT3/NF-κB/SETDB2 axis in normal and diabetic wound macrophages.

Given our prior findings that SETDB2 is decreased in T2D wound macrophages combined with our present data that identifies, to our knowledge, a novel role for STAT3 regulation of SETDB2, we examined whether STAT3 regulation of SETDB2 was altered in diabetic wound macrophages. To compare *STAT3* expression between healthy and T2D macrophages, we analyzed scRNA-Seq in human wound tissue isolated from patients without diabetes and those with T2D (19). We found that *STAT3* expression was significantly increased in macrophages from human T2D wounds versus controls (Figure 5A). To model the pathologic process of diabetic wound healing, we routinely use the diabetic-induced obese (DIO) mouse model, which, while physiologically closely reflects a prediabetic state, has been well established by our lab and others to model the pathologic effects of T2D on wound healing (35). Thus, we isolated wound macrophages from DIO and normal diet (ND) mice and then treated macrophages with IFN-β, which we previously identified as an upstream regulator of SETDB2. Compared with ND macrophages, DIO macrophages exhibited increased expression of *Stat3* and *Nfkb/c-Rel* (Figure 5, B and C). This was also verified at the protein level by performing Western blotting of whole wounds and wound macrophages isolated from ND and DIO mice at different time points (Supplemental Figure 6, A and B). Since we showed that STAT3 inhibited the RELA/SETDB2 interaction in normal macrophages, we next asked if increased STAT3 in diabetic macrophages disrupted the RELA/SETDB2 interaction, perhaps leading to increased NF-κB–mediated inflammation due to decreased SETDB2 at NF-κB–dependent promoters. To test this, we performed coimmunoprecipitation of Stat3 with endogenous Setdb2 in whole wound lysates from ND and DIO mice obtained day 5 after wounding. We observed increased binding of Stat3 to Setdb2 in DIO wounds compared with ND wounds (Figure 5D). To test whether the differences in dysregulated Stat3 were due to increased activation, we measured phosphorylated Stat3 in ND and DIO macrophages since Stat3 is phosphorylated in its active form. Basal Stat3 phosphorylation was not majorly different between ND and DIO BMDMs, indicating that differences in Stat3/RelA binding were most likely due to increased *Stat3* expression rather than Stat3 activation and/or affinity to Setdb2 (Supplemental Figure 7). Finally, to test whether Stat3 deletion in macrophages could improve wound healing in the diabetic setting, we performed wound curve analysis in DIO mice with macrophage-specific deletion of Stat3 (*Stat3^fl/fl^ Lyz2^Cre+^)* compared with Cre-negative controls. Indeed, Stat3 deletion in macrophages drastically improved early wound healing in DIO mice (Figure 5E). Taken together, these results demonstrate that *STAT3* is increased in human and murine diabetic wound macrophages and exhibits a stronger interaction with SETDB2 in diabetic wounds, and we established that deletion of STAT3 in macrophages improves diabetic wound healing in vivo (Figure 6).

## Discussion

In this paper, we sought to determine the transcription mechanisms regulating *SETDB2* expression and activity in wound macrophages. This is important given the critical and dynamic role that SETDB2 plays in regulating inflammatory gene transcription in wound repair. To determine the global transcriptional effect of SETDB2 in macrophages during wound healing, we performed parallel RNA-Seq and ATAC-Seq in murine wound macrophages deficient in Setdb2 and identified upregulation of NF-κB–dependent inflammatory genes as well as increased chromatin accessibility in several of these genes, respectively. Next, we identified transcription mechanisms regulating SETDB2, which consisted of identifying both TFs regulating *SETDB2* expression and identifying SETDB2 binding partners that influenced its transcription activity. In our analysis of the human *SETDB2* promoter, we identified the TF STAT3 as a potent transcriptional regulator of *SETDB2* expression in macrophages. To investigate the downstream transcriptional effects of STAT3 in wound repair, we performed RNA-Seq on Stat3-deficient murine wound macrophages and demonstrated increased expression of inflammatory genes, which were within pathways that overlapped those regulated by SETDB2, as determined by our RNA-Seq analysis. In GST pulldown and immunoprecipitation experiments, we found that STAT3 and RELA bound SETDB2. Finally, we found that STAT3 expression and physical interaction with SETDB2 was increased in diabetic macrophages and macrophage-specific deletion of STAT3 improved wound healing in diabetic mice.

To our knowledge, this is the first report to identify interactions between SETDB2, NF-κB, and STAT3, thereby defining a critical axis between these transcriptional regulators. Additionally, using human samples and transgenic mice, we demonstrate that STAT3 was required for *SETDB2* expression but also paradoxically limited SETDB2 binding to the RELA NF-κB component and associated NF-κB–dependent gene promoters. We are the first to our knowledge to identify the global downstream transcriptional profiles of wound macrophages absent in SETDB2 or STAT3 using ATAC-Seq and RNA-Seq. Finally, we demonstrate that genetic deletion of STAT3 in macrophages improves normal and diabetic wound healing at early time points.

Our previous work showed that *SETDB2* expression in macrophages was positively regulated by IFN-β/JAK/STAT1 signaling. While our prior work demonstrated that STAT1 regulated *SETDB2* transcription, we asked whether other STAT family members were also required. We believe the present study is highly novel in its expansion on our previous work, as we use a detailed analysis of the *SETDB2* promoter to identify additional TFs regulating *SETDB2* expression. In contrast to our previous work, we specifically interrogated upstream transcriptional regulators that were differentially expressed between diabetic and healthy macrophages. Indeed, our data support STAT3 as the predominant regulator of *SETDB2* expression. We demonstrate here using our scRNA-Seq data that *STAT3* expression was selectively upregulated in human diabetic wound macrophages compared with control. We also identified several STAT3 binding sites in the human *SETDB2* promoter, which was confirmed by ChIP of STAT3 at the *SETDB2* promoter. Additionally, macrophage-specific deletion of STAT3 in vivo significantly decreased *SETDB2* expression in macrophages. Our data that STAT3 increases *SETDB2* expression to ultimately decrease inflammatory gene expression are consistent with evidence from other labs that STAT3 can repress gene expression and provide another mechanism of the less-studied noncanonical function of STAT3 (36, 37).

While decreased *SETDB2* expression drives the aberrant proinflammatory macrophage phenotype in the diabetic wound, there have been no studies that have examined the specific transcription mechanisms regulating SETDB2 function and whether altered upstream transcriptional regulation of SETDB2 activity contributes to disease. Numerous studies in macrophages as well as other cell types have demonstrated that TFs can bind to epigenetic enzymes and other TFs to cooperatively regulate their function and alter disease phenotype (38–40). Here, we show that SETDB2 interacts with the STAT3 and NF-κB component RELA. Specifically, STAT3 inhibits the association between SETDB2-, RELA-, and NF-κB–dependent promoter regions, thereby leading to decreased H3K9me3 and increased inflammatory gene expression. Thus, in addition to its canonical role in regulating the proinflammatory macrophage phenotype via the IFN-β/JAK/STAT pathway, STAT3 also promotes inflammation by directly regulating the physical association between SETDB2 and NF-κB. However, while STAT3 inhibits SETDB2 function, it is required for *SETDB2* expression in normal wound macrophages. Therefore, tight control of SETDB2 levels and activity by STAT3 modulates the macrophage gene program during wound healing. We also demonstrate that *STAT3* is increased in diabetic macrophages and binds more strongly to SETDB2. Furthermore, macrophage-specific deletion of STAT3 in diabetic mice results in improved wound healing, which is more pronounced in early time points of wound repair. This suggests that, in the acute phase of diabetic wound healing, increased STAT3 may interfere with the SETDB2/NF-κB interaction and tissue repair. In this manner, STAT3 titrates SETDB2- and NF-κB–dependent gene transcription in macrophages during wound healing, and this axis is perturbed in the diabetic setting. Pharmacologic inhibition of the STAT3/SETDB2 interaction at specific, perhaps earlier, time points in the acute phase of tissue repair may thus improve diabetic wound healing.

While our results identify similar overlapping DEGs in IFN-regulated pathways between SETDB2- and STAT3-KO wound macrophages, the specific DEGs were not completely identical. This indicates that the overall effect on macrophage phenotypic switching by SETDB2 and STAT3 may be due to a combinatorial effect of these transcriptional regulators, rather than 1 specific gene set. Furthermore, the specific role of SETDB2 or STAT3 is likely time dependent throughout wound healing. For example, STAT3 may regulate the transcription activity of SETDB2 and downstream inflammatory genes at a more optimal time point than day 5, which is when the CD11b^+^ macrophages were harvested from wounds. Additionally, these downstream transcriptional profiles are likely reflective of both the IFN-β and NF-κB pathways, which can occur simultaneously and can have opposing and overlapping roles. For example, although IFN-β leads to upregulation of *SETDB2* expression, which leads to silencing of NF-κB–dependent genes, increased IFN-β/STAT3 limits the NF-κB/RELA/SETDB2 association, thereby resulting in heightened inflammation. Thus, the specific outcomes of these 2 pathways on macrophage phenotype are likely time and context specific. Furthermore, we acknowledge that the current approach of using Ly6C^+^ and CD11b^+^ as markers to identify the macrophage subtype more realistically captures a spectrum of myeloid cells, one that is likely representative of multiple subpopulations. However, we have attempted to overcome this limitation by subsetting CD11b^+^ into Ly6C^hi^ and Ly6C^lo^ populations, since we have demonstrated previously that the majority of the macrophage population after wounding arises from the infiltrating Ly6C^hi^ monocyte population, rather than the Ly6C^lo^ or tissue-resident macrophage populations (41).

In conclusion, we identified that SETDB2 functions with NF-κB to regulate the proinflammatory transcriptional program in macrophages during wound healing by directly targeting NF-κB–dependent gene regions to influence chromatin accessibility. We demonstrate that STAT3 transcriptionally regulates SETDB2 via 2 pathways. First, STAT3 increases *SETDB2* expression, which decreases NF-κB–driven inflammation. Simultaneously, STAT3 inhibits SETDB2 binding to RELA, leading to derepressed (i.e., increased) inflammation. In diabetic wounds, *STAT3* expression and binding to SETDB2 are increased, and STAT3 deletion in macrophages improves wound healing. By defining what we believe to be a novel STAT3/SETDB2/NF-κB axis, we have identified several potential targets to treat normal wound healing as well as nonhealing diabetic wounds in a cell-specific and time-dependent manner. For example, immunomodulators that increase *STAT3* expression (and thus *SETDB2* expression) during normal wound healing may be useful. In contrast, in diabetic wounds in which the STAT3/NF-κB axis is perturbed, inhibiting STAT3 during early diabetic wound healing may increase SETDB2 localization to active inflammatory gene regions, thereby inhibiting pathologic inflammation and improving tissue repair.

## Methods

### Sex as a biological variable.

Male mice were used in this study because female mice on a DIO model are less likely to develop the diabetic phenotype. Despite this, our findings are applicable to both sexes, as diabetic wound complications are present in both sexes.

### Mice.

All mice were maintained at the University of Michigan Biomedical Sciences and Research Building in the Unit for Laboratory and Animal Medicine (ULAM). C57BL/6 mice (RRID: IMSR_JAX:000664) were obtained at 6–7 weeks age from The Jackson Laboratory and maintained in breeding pairs in the ULAM facilities. *Setdb2^fl/fl^* mice were created as previously published by our lab (42). *Setdb2^fl/fl^* mice were bred with B6.129P2-Lyz2^tm1(Cre)Ifo/J^ mice and *Gt(ROSA)26Sor^tm4(ACTB–tdTomato,–EGFP)Luo^/J* (*mTmG*) from The Jackson Laboratory to generate *Setdb2^fl/fl^ Lyz2^Cre^* mice deficient of *Setdb2* in monocytes, macrophages, and granulocytes on the mTmG reporter background (43). *Stat3^fl/fl^* mice were cryopreserved and obtained from The Jackson Laboratory and crossed to B6.129P2-Lyz2^tm1(Cre)Ifo/J^ mice to generate *Stat3^fl/fl^ Lyz2^Cre^* mice with myeloid-specific KO of Stat3. Numbers of mice used per experiment can be found in the figure legend of each corresponding experiment. Mice were genotyped regularly after birth with custom primers. All mice were maintained in breeding pairs at the ULAM facilities. Male C57BL/6 mice were maintained on a standard normal rodent diet (13.5% kcal saturated fat, 28.5% protein, 58% carbohydrate; Lab Diet) or standard high-fat diet (60% kcal saturated fat, 20% protein, 20% carbohydrate; Research Diets Inc.) for 12–18 weeks to induce the DIO model of T2D mellitus (35, 44). After 12–18 weeks, DIO mice developed obesity and insulin resistance with fasting blood sugars in the mid-200s and elevated insulin levels. All DIO and control animals underwent procedures at 20–32 weeks of age with IACUC approval. For these experiments, only male mice were used, as female mice are less likely to develop DIO.

### Wound healing assays.

Mice were appropriately anesthetized and hair was removed with a hair-removal agent (Veet). Full-thickness 4 mm punch biopsies were made in the dorsum of each mouse (2 wounds per mouse for wound curves; 4 wounds per mouse for downstream cell isolation). Wounds were measured beginning day 0 through day 7. Wounds were harvested for macrophage isolation per previously established protocol at the given time point. At the conclusion of the experiment, mice were euthanized.

### Macrophage isolation from wounds.

Wounds were collected in RPMI with glutamine and antibiotics and minced into small pieces before being incubated with liberase and DNase for 30 minutes. Wounds were passed through a 100 μm strainer to generate a single-cell suspension. Cells were incubated with biotin-labeled anti-CD3 (BioLegend, 100304), anti-CD19 (BioLegend, 115504), and anti-Ly6G (BioLegend, 127604), followed by incubation with streptavidin RapidSpheres (Stemcell Technologies, 19860A). Flow-through was then incubated with anti-CD11b and Dextran RapidSpheres (Stemcell Technologies, 18970A) to isolate the non-neutrophil, nonlymphocyte, and CD11b^+^ cells. Cells were either collected directly in TRIzol (Invitrogen) for RNA extraction or plated appropriately for specific downstream experiments.

### Western blotting.

Cells were lysed in RIPA buffer plus protease and phosphatase inhibitors and 1 mM DTT. Lysates were normalized by total protein concentration, sample buffer was added, and then samples were boiled for 5 minutes. In total, 10–50 μg of protein was loaded on an SDS-PAGE gel and run, and proteins were transferred to nitrocellulose membranes and blocked in either 3% BSA or 5% nonfat dry milk. Blocked membranes were incubated with primary antibodies in 1% BSA or 5% milk. The following antibodies and concentrations were used: SETDB2 antibody (Invitrogen, PA5-110357; 1:500 dilution), p65/RELA antibody (Cell Signaling Technology, 8242; 1:1,000 dilution), and STAT3 antibody (Cell Signaling Technology, 9132; 1:1,000 dilution). Membranes were washed and incubated with species-specific HRP-linked secondary antibodies, washed, and developed. For densitometry results, Western blots were analyzed using ImageJ software (NIH).

### Immunoprecipitations.

Cells were lysed as above for Western blotting. In total, 5 μg of SETDB2 antibody was linked to Protein G Dynabeads, and then linked antibody beads were added to lysate and rotated overnight at 4°C. Immunoprecipitated complexes were washed and eluted in sample buffer by boiling. Samples were processed similar to Western blotting protocol detailed above using appropriate antibodies.

### GST-pulldown assays.

Recombinant GST-SETDB2 fusion protein was purchased from MilliporeSigma (catalog SRP5261). GST Sepharose beads were prepared by washing 2× with PBS. In total, 50 μL of beads were incubated with 20 μg of GST-SETDB2 fusion protein or GST negative control for 1 hour at 4°C with rotation. GST-fusion protein beads were then washed 1× with PBS and then incubated with 500 μg BMDM lysate overnight at 4°C with rotation. The following day, beads were washed 3× in RIPA buffer and 1× in TBS. Samples were boiled in 30 μL 2× sample buffer for 5 minutes and then run on SDS-PAGE gel for Western blotting.

### Luciferase assays.

The human *SETDB2* promoter was amplified using PCR from human genomic DNA template and then cloned into pGL3 empty vector (EV). Correct sequence was confirmed by Sanger sequencing. BMDMs were plated at 2 × 10^5^ to 3 × 10^5^ per well in 24-well plates and transfected with 0.5 μg pGL3-*SETDB2* promoter or pGL3-EV per well. Cells were transfected for 48 hours, and then luciferase assays were performed at that time using Steady-Glo Luciferase Kit (Promega, E2520) according to manufacturer’s instructions.

### ChIP experiments.

After appropriate treatment, macrophages were fixed with 0.7% PFA and then quenched with 0.125 M glycine. Cells were rinsed and collected in 1× PBS. ChIP was performed using the Abcam ChIP kit (catalog ab500) according to manufacturer’s instructions. In total, 4 μL ChIP sample was used in downstream qPCR.

### FACS.

Wounds were harvested on day 5 from mice that had undergone 4 mm punch biopsies. Wounds were digested into a single-cell suspension as previously described, and then MACS was used to isolate macrophages (CD3^–^CD19^–^NK1.1^–^Ly6G^–^CD11b^+^). Cells were then surface stained with an Anti-Ly6C antibody (BioLegend, 128035, 1:400 dilution). Samples were sorted by FACS on a FACSAria III Flow Sorter. FACS was performed with FACSDiva Software (BD Biosciences), analysis was performed using FlowJo software version 10.0 (Tree Star Inc.), and data were compiled using Prism software (GraphPad). All populations were routinely back-gated to verify gating and purity. In the case of SETDB2 experiments, macrophages were isolated from *Setdb2^fl/fl^ Lyz2*^Cre^ *mTmG* mice, and CD3^–^CD19^–^Ly6G^–^CD11b^+^Ly6C^+^GFP^+^ cells were separated into CD11b^+^Ly6C^hi^ and CD11b^+^Ly6C^lo^ monocyte/macrophage populations, which were used for downstream ATAC-Seq and RNA-Seq analyses.

### ATAC-Seq.

Wound macrophages were harvested from mice at day 5 as described above using MACS, and nuclei were isolated as described previously (45). After counting, 50,000 nuclei per sample were resuspended in the appropriate volume, spun down, and collected for use in downstream transposition reaction per modified ENCODE protocol (46). Data were obtained and processed according to the ENCODE pipeline and then converted to readable format as similarly described (19). Briefly, minimum read length prior to trimming was 45 bp. Paired-end sequencing was performed using NovaSeq (Illumina), and sequences were mapped to the genome.

### RNA-Seq.

Day 5 wounds were harvested from *Setdb2^fl/fl^ Lyz2^Cre^*, *Stat3^fl/fl^ Lyz2^Cre^*, and Cre-negative littermate control mice, digested, and underwent negative selection for CD3, CD19, and Ly6G lineage markers. Remaining cells were surface stained and sorted by FACS into CD11b^+^Ly6C^hi^ and CD11b^+^Ly6C^lo^ populations. RNA isolation was performed using a RNeasy Kit (Qiagen) with DNase digestion. Library construction and analysis of reads were performed as described previously (41). Briefly, reads were trimmed using Trimmomatic and mapped using HiSAT2 (47, 48). Read counts were performed using the feature counts option from the subRead package followed by the elimination of low reads, normalization, and differential gene expression using edgeR (49, 50). Differential expression was performed on mapped reads using the taqwise dispersion algorithm in edgeR.

### Human wound isolation.

Biopsies from human diabetic wounds (*n* = 5) and nondiabetic skin samples (*n* = 5) were collected. Control nondiabetic samples were obtained from amputation specimens from patients who had an HbA1c < 7%. The samples used for scRNA-Seq from patients with diabetes were from patients at an average age of 60 years (HbA1c > 7%) who had hypertension, hyperlipidemia, and coronary artery disease. In the samples from patients without diabetes, the average age was 70 years, with half the patients having hypertension, hyperlipidemia, and coronary artery disease. Wounds were obtained from the specimens using an 8 mm punch biopsy and were processed for downstream analysis.

### scRNA-Seq analyses.

Generation of single-cell suspensions for scRNA-Seq was performed in the following manner as described by our group previously (19). Briefly, skin was harvested via punch biopsy from diabetic and nondiabetic control patient wounds. The epidermis and dermis layers were isolated and digested separately. Epidermal and dermal cells were strained and then combined in a 1:1 ratio for scRNA-Seq by the University of Michigan Advanced Genomics Core on the 10X Genomics Chromium System. Libraries were sequenced on the Illumina NovaSeq 6000 sequencer. NovaSeq was used as the sequencing platform to generate 151 bp paired-end reads. We conducted adapter trimming and quality control procedures as described previously (51). The reads were then mapped using STAR (52) to build human GRCh37, and gene expression levels were quantified and normalized by HTSeq (53) and DESeq2 (54), respectively. Negative binomial models in DESeq2 were used to conduct differential expression analysis. To increase the sample size of the control samples, we used the skin biopsies obtained from our previous study (55). Data processing, including quality control, read alignment, and gene quantification, was conducted using the 10X Genomics Cell Ranger software. Seurat was then used for normalization, data integration, and clustering analysis (56). All clustered cells were mapped to corresponding cell types by matching cell cluster gene signatures with putative cell type–specific markers.

### Statistics.

GraphPad Prism software (RRID:SCR_002798) version 9.2.0 was used to record and analyze the data. Statistical significance between 2 groups was determined using a 2-tailed Student’s *t* test and between multiple groups using ANOVA (1- or 2-way, dependent on experiment), where appropriate. All *P* values less than or equal to 0.05 were considered significant.

### Study approval.

All experiments using human samples were conducted after informed consent from patients and approved by the IRB at the University of Michigan (no. HUM00098915); experiments were conducted in accordance with the principles in the Declaration of Helsinki. Mouse experiments were conducted with approval from our IACUC (protocol no. PRO00009811).

### Data availability.

For bulk RNA-Seq and scRNA-Seq data accession, the accession numbers include GSE154556, GSE154557, and GSE274112 (Gene Expression Omnibus). See the Supporting Data Values file for individual data points for each figure.

## Author contributions

KDM designed the research studies, performed the experiments, acquired the data, analyzed the data, wrote, and edited the manuscript. AD designed the research studies, performed the experiments, acquired the data, and analyzed the data. LCT, QL, and RW acquired and analyzed the scRNA-Seq, RNA-Seq, and ATAC-Seq data. SJW, JYM, WJM, ADJ, COA, FMD, ATO, ECB, TMB, KB, and ZA aided in experimental design and approach and performed experiments. JS aided in experimental design and approach. FMD, JG, and KAG aided in experimental design, data analysis, and edited the manuscript.

## Supplementary Material

Supplemental data

Unedited blot and gel images

Supplemental table 1

Supplemental table 2

Supplemental table 3

Supporting data values

## Version 1 10/22/2024

Electronic publication
