# Supplementary material for: The STAT3/SETDB2 axis dictates NF-κB–mediated inflammation in macrophages during wound repair
Source: JCI Insight. 2024 Oct 22;9(20):e179017. doi: 10.1172/jci.insight.179017 (PMC11530128; doi:10.1172/jci.insight.179017)
Supplement: Supplemental data [file jciinsight-9-179017-s230.pdf]

Supplemental Figures

Supplemental Figure 1

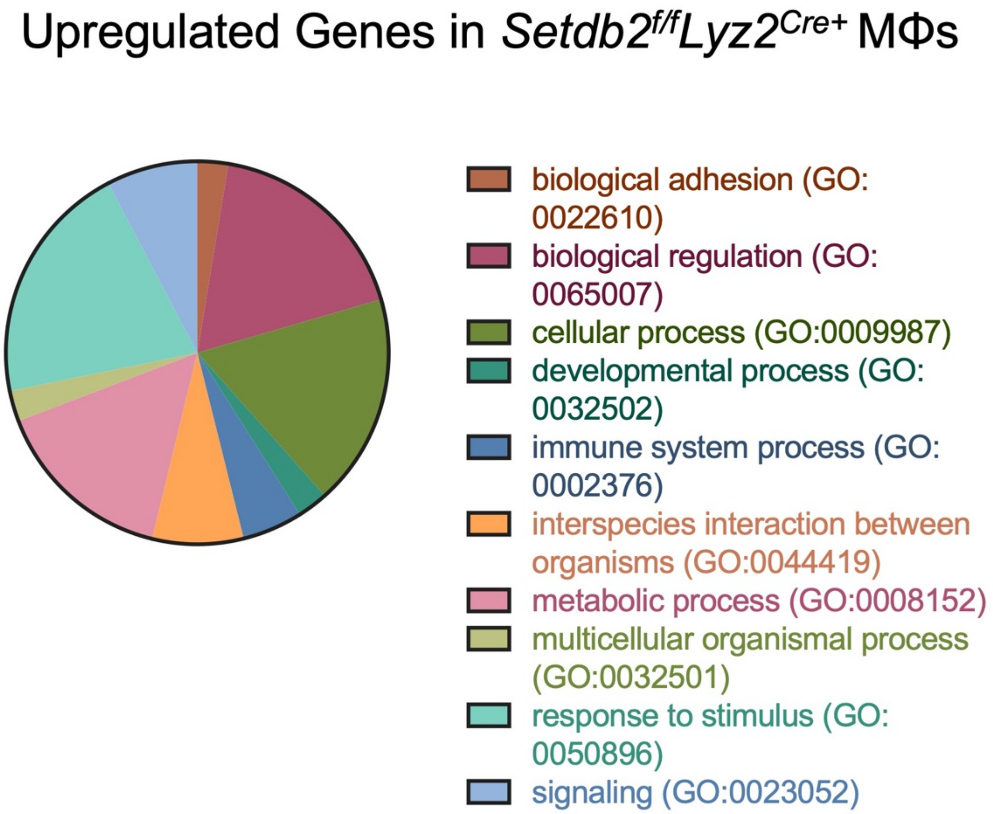

Supplemental Figure 2

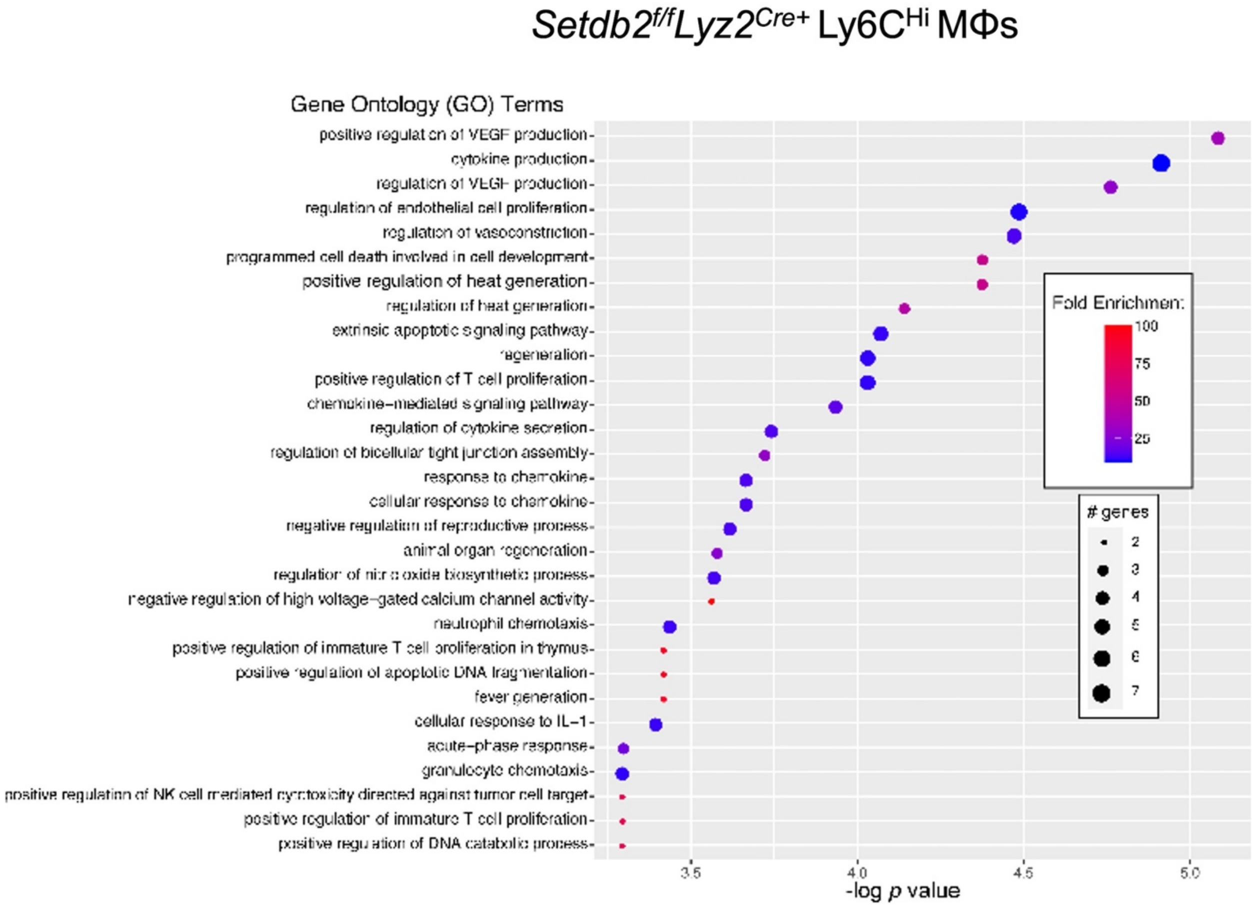

Supplemental Figure 3

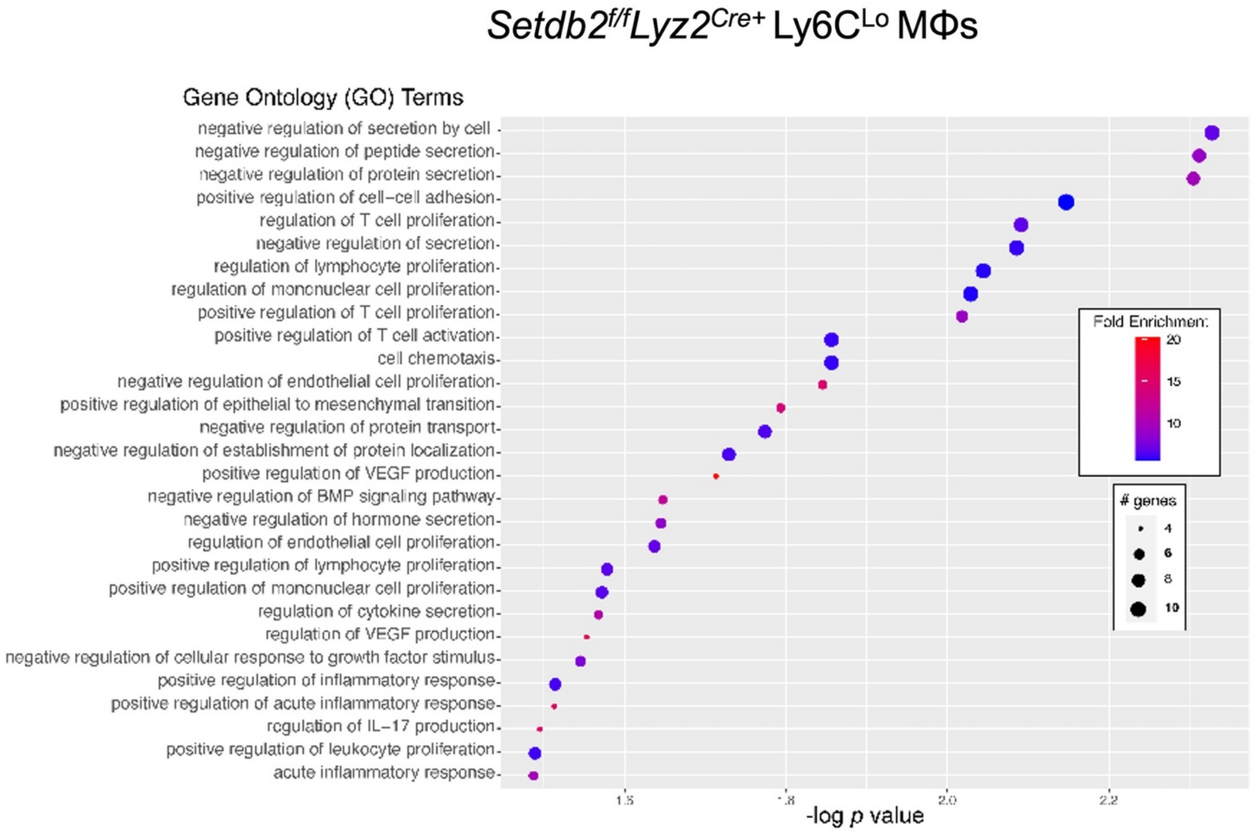

Supplemental Figure 4

NFκB-regulated gene networks in *Setdb2*$^{f/f}$*Lyz2*$^{Cre+}$ Ly6C^{Hi} MΦs

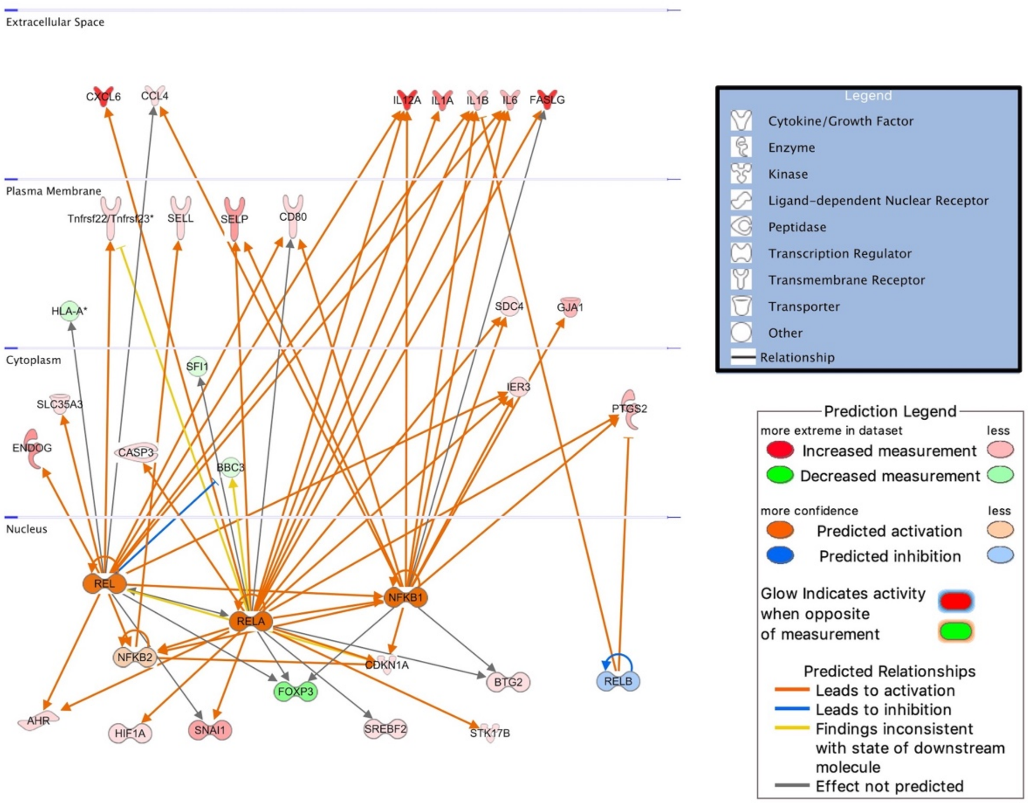

Supplemental Figure 5

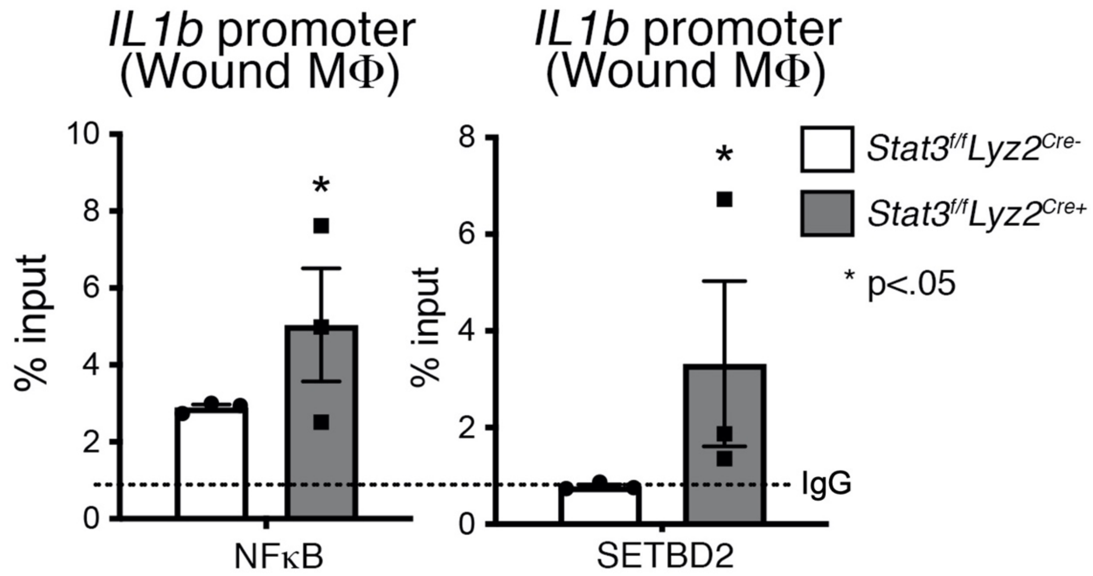

Supplemental Figure 6

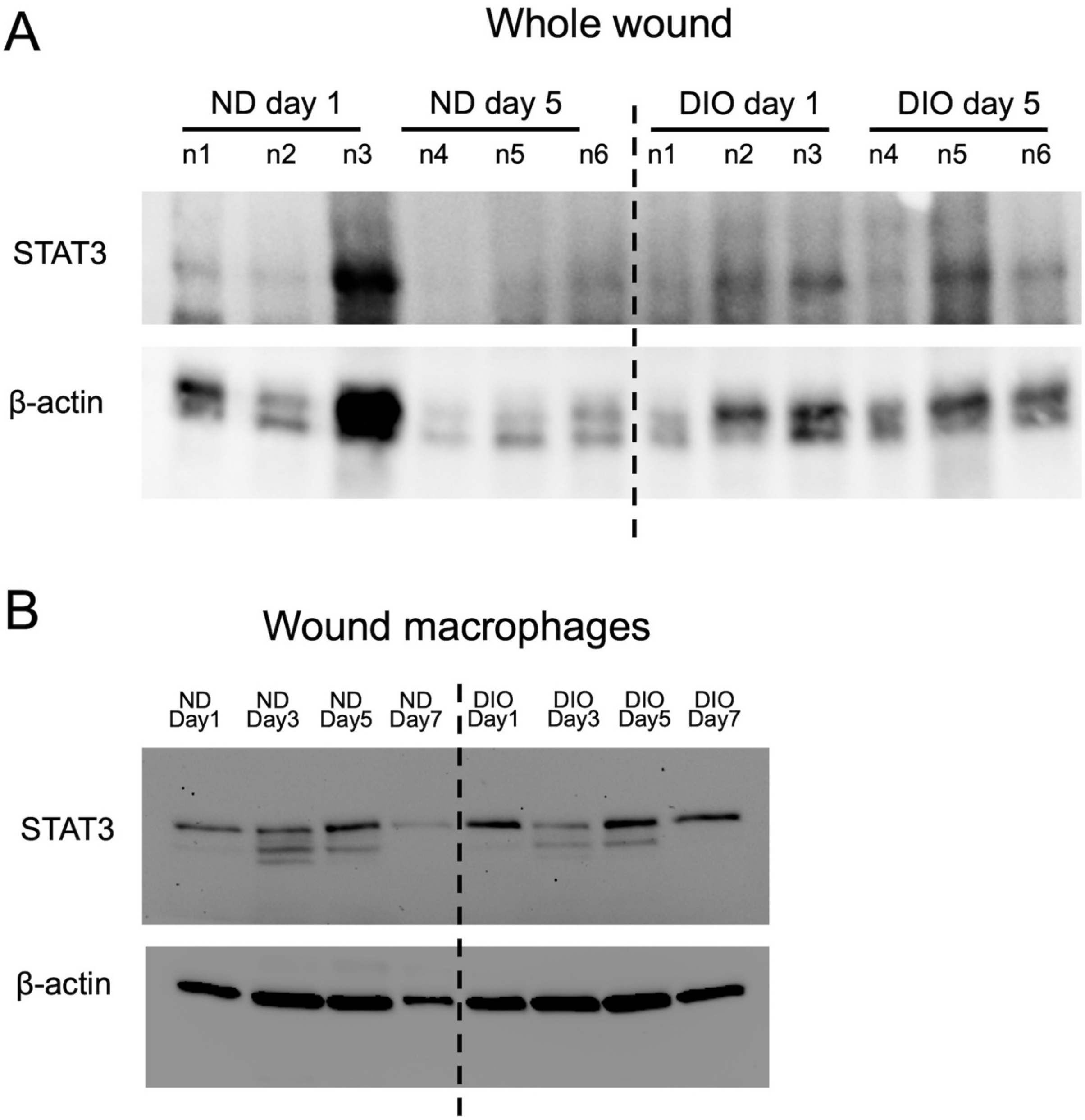

Supplemental Figure 7

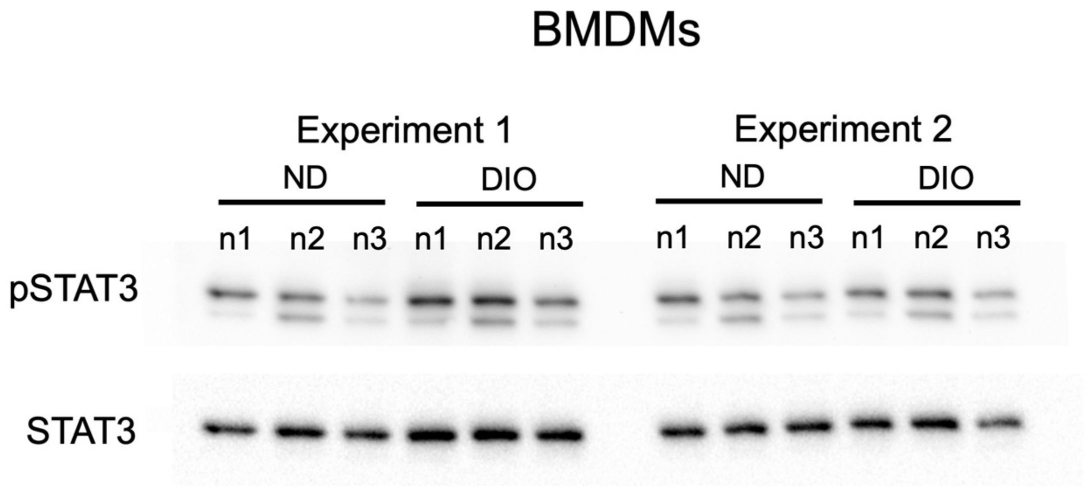

Supplemental Legends

Supplemental Figure 1. SETDB2 regulates various biologic and cellular processes in macrophages. Wound macrophages were isolated from *Setdb2*$^{flox/flox}$*Lyz2*^{Cre}*mTmG* murine wounds on day 5 post-wounding, sorted into CD3$^-$CD19$^-$NK1.1$^-$Ly6G$^-$CD11b$^+$Ly6C^{Hi} and CD3$^-$CD19$^-$NK1.1$^-$Ly6G$^-$CD11b$^+$Ly6C^{Lo} populations, and then analyzed for gene expression by RNA-seq. GO analysis was performed on upregulated genes.

Supplemental Figure 2. Regulation of downstream pathways by SETDB2 in Ly6CHi macrophages. Wound macrophages were isolated from *Setdb2*$^{flox/flox}$*Lyz2*^{Cre}*mTmG* murine wounds on day 5 post-wounding, sorted into CD3$^-$CD19$^-$NK1.1$^-$Ly6G$^-$CD11b$^+$Ly6C^{Hi} and CD3$^-$CD19$^-$NK1.1$^-$Ly6G$^-$CD11b$^+$Ly6C^{Lo} populations, and then analyzed for gene expression by RNA-seq. GO analysis was performed in the Ly6CHi population.

Supplemental Figure 3. Regulation of downstream pathways by SETDB2 in Ly6CLo macrophages. Wound macrophages were isolated from *Setdb2*$^{flox/flox}$*Lyz2*^{Cre}*mTmG* murine wounds on day 5 post-wounding, sorted into CD3$^-$CD19$^-$NK1.1$^-$Ly6G$^-$CD11b$^+$Ly6C^{Hi} and CD3$^-$CD19$^-$NK1.1$^-$Ly6G$^-$CD11b$^+$Ly6C^{Lo} populations, and then analyzed for gene expression by RNA-seq. GO analysis was performed in the Ly6CLo population.

Supplemental Figure 4. Regulation of downstream pathways by SETDB2 in Ly6CLo macrophages. Wound macrophages were isolated from *Setdb2*$^{flox/flox}$*Lyz2*^{Cre}*mTmG* murine wounds on day 5 post-wounding, sorted into CD3$^-$CD19$^-$NK1.1$^-$Ly6G$^-$CD11b$^+$Ly6C^{Hi} and CD3$^-$CD19$^-$NK1.1$^-$Ly6G$^-$CD11b$^+$Ly6C^{Lo} populations, and then analyzed for gene expression by RNA-seq. Pathways analysis was performed to identify NFkB-related gene networks in the Ly6CHi population.

Supplemental Figure 5. STAT3 inhibits NFkB-SETDB2 binding at the *Il1b* promoter. ChIP-qPCR for NFkB (RELA) and SETDB2 at the *Il1b* promoter in *Stat3*$^{flox/flox}$*Lyz2*$^{Cre+}$ versus cre-negative macrophages (CD3$^-$CD19$^-$NK1.1$^-$Ly6G$^-$CD11b$^+$) isolated from *Stat3*$^{flox/flox}$*Lyz2*$^{Cre+}$ *and* littermate cre-negative controls on day 5 post-wounding (n=4-6 mice per group). All data are representative of 2-4 independent experiments. *$p < 0.05$. Data are presented as the mean ± SEM. Data were first analyzed for normal distribution, and if data passed the normality test, two-tailed Student's t test was used.

Supplemental Figure 6. STAT3 expression in ND and DIO wound macrophages. A) Wounds were harvested day 5 post-wounding ND and DIO mice, snap frozen, and proteins extracted in RIPA buffer. Protein lysates were run on SDS-PAGE gels for Western blotting and then probed for STAT3. B) CD3$^-$CD19$^-$NK1.1$^-$Ly6G$^-$CD11b$^+$ cells were isolated from ND and DIO murine wounds by MACS on day 5 post-wounding. Cells were collected in RIPA buffer and lysates were run on SDS-PAGE gels for Western blotting and then probed for STAT3.

Supplemental Figure 7. p-STAT3 in ND and DIO macrophages. CD3$^-$CD19$^-$NK1.1$^-$Ly6G$^-$CD11b$^+$ cells were isolated from ND and DIO murine wounds by MACS on day 5 post-wounding. Cells were collected in RIPA buffer and lysates were run on SDS-PAGE gels for Western blotting and then probed for phospho-STAT3, and then blots were stripped and reprobed for total STAT3.

Supplemental Table 1. List of DEGs generated from RNA-seq of CD3$^-$CD19$^-$NK1.1$^-$Ly6G$^-$CD11b$^+$Ly6C^{Hi} wound macrophages from *Setdb2*$^{flox/flox}$*Lyz2*^{Cre}*mTmG* mice.

Supplemental Table 2. Complete list of differentially regulated gene positions generated from ATAC-seq of $CD3^-CD19^-NK1.1^-Ly6G^-CD11b^+Ly6C^{Hi}$ wound macrophages from *Setdb2*$^{flox/flox}$*Lyz2*^{Cre}*mTmG* mice.

Supplemental Table 3. List of DEGs generated from RNA-seq of $CD3^-CD19^-NK1.1^-Ly6G^-$ $CD11b^+Ly6C^{Hi}$ wound macrophages from from *Stat3*$^{flox/flox}$*Lyz2*^{Cre} mice.

Summary Figure. Dysregulated STAT3 modulates SETDB2-NFkB binding and transcriptional regulation of inflammatory genes in diabetic wound healing. Illustration of negative regulation of inflammatory gene expression by SETDB2 in normal wound macrophages versus dysregulated wound healing in diabetic macrophages, in which STAT3 is increased and aberrantly binds SETDB2 to prevent its association with NFkB at inflammatory gene promoters.
