# Supplementary material for: The STAT3/SETDB2 axis dictates NF-κB–mediated inflammation in macrophages during wound repair
Source: JCI Insight. 2024 Oct 22;9(20):e179017. doi: 10.1172/jci.insight.179017 (PMC11530128; doi:10.1172/jci.insight.179017)

Unedited blots

R2 Mangum et al.

Full unedited gel for Figure 2H

Stat3

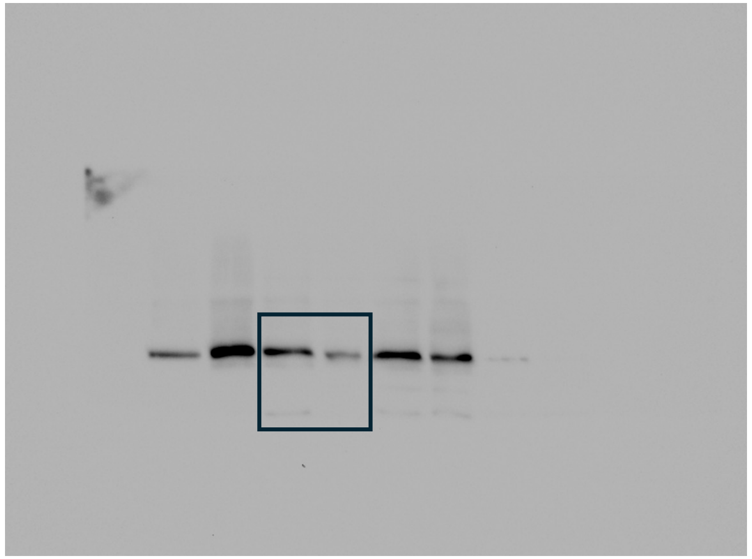

β-actin

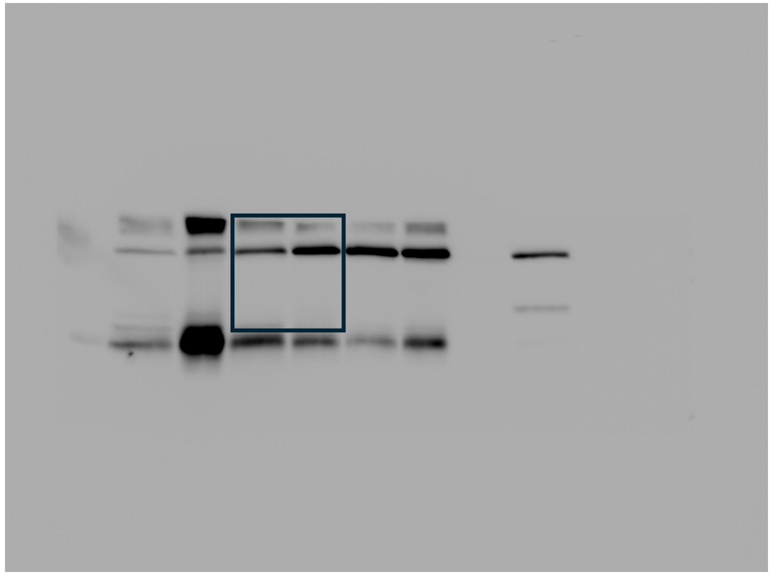

Full unedited gel for Figure 3B

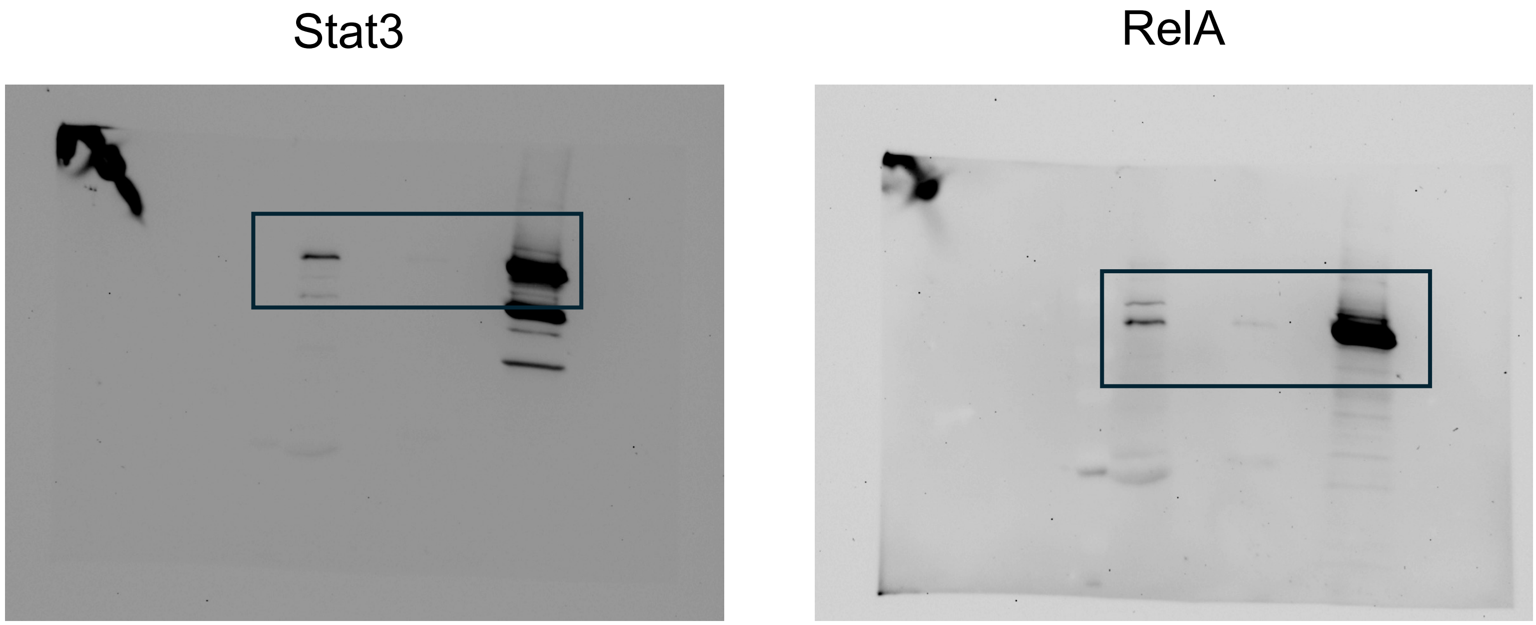

Full unedited gel for Figure 3C

RelA

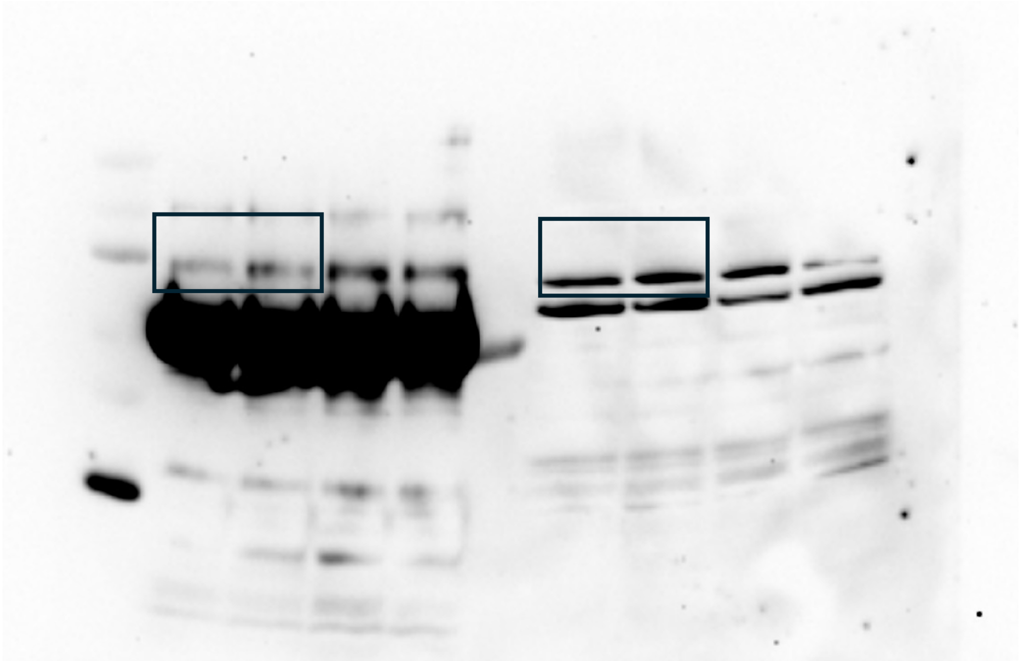

Full unedited gel for Figure 3D

RelA

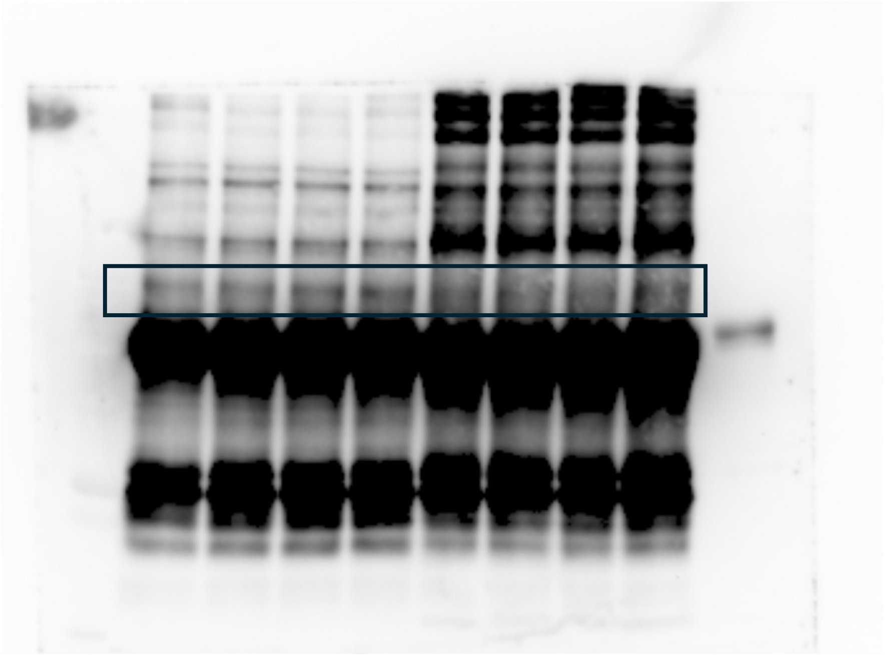

Setdb2

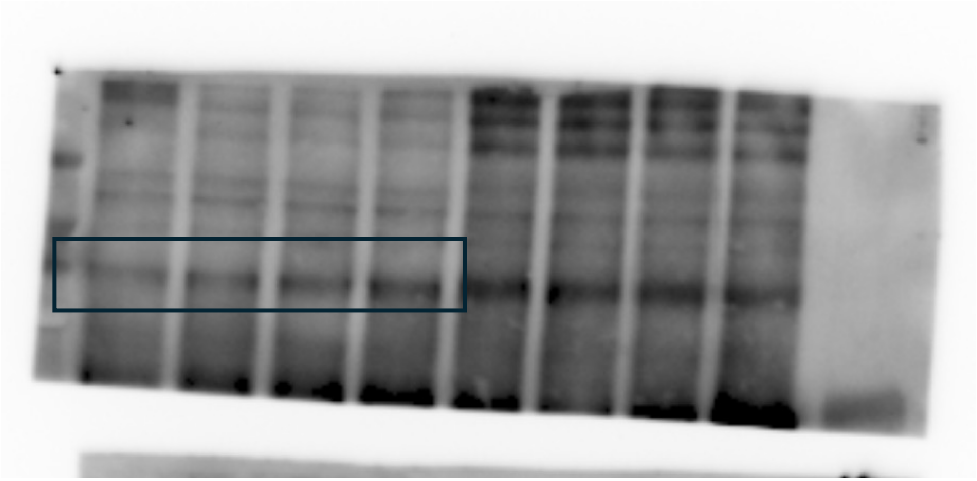

Full unedited gel for Figure 5D

Stat3 (IP)

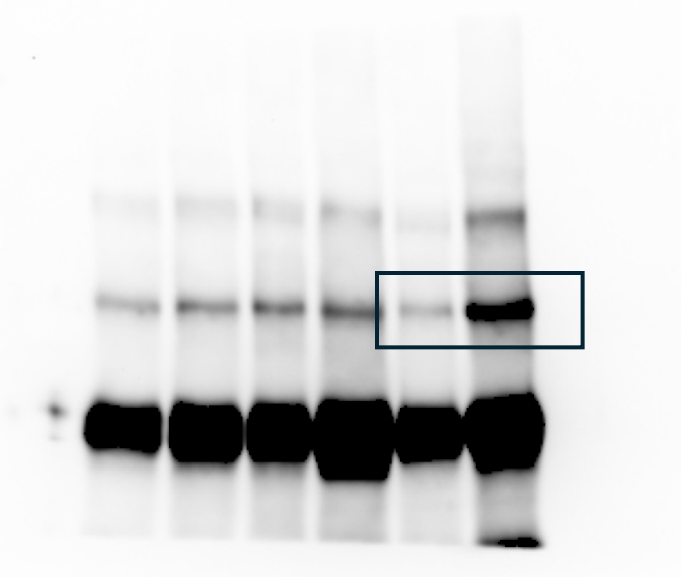

Stat3 (input)

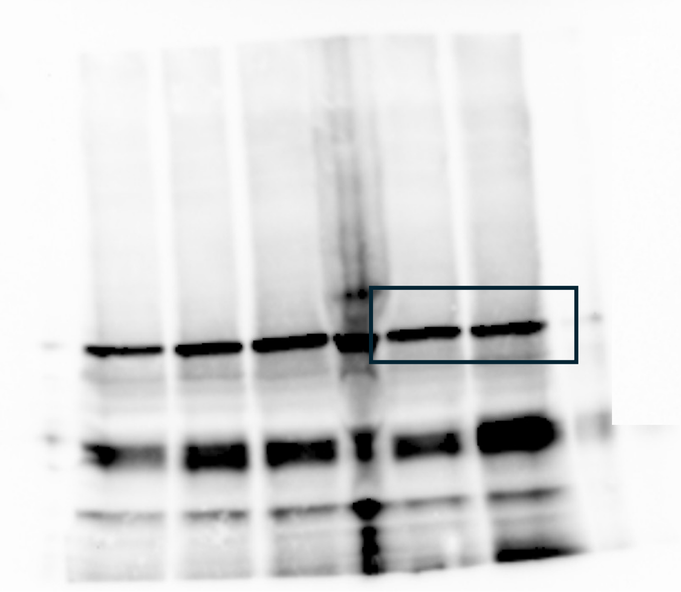

Setdb2 (IP)

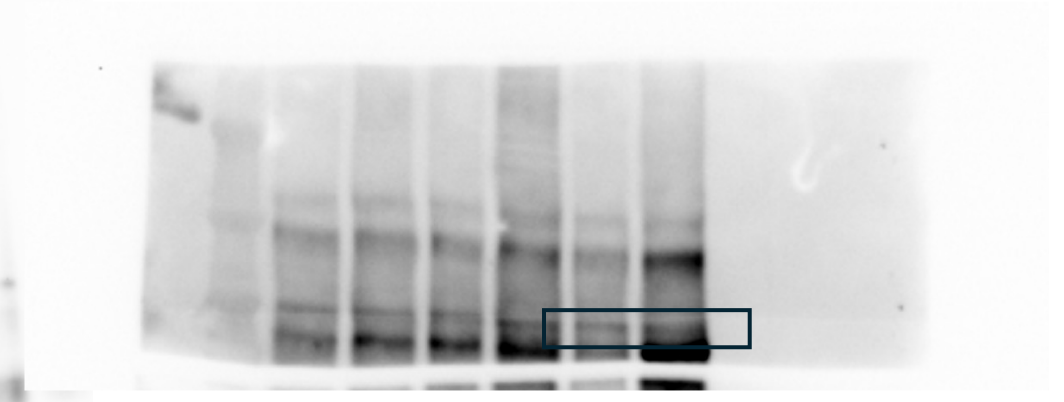

Supplement: Unedited blot and gel images [file jciinsight-9-179017-s231.pdf]
